# Leveraging Reaction Heterogeneity in Bimodal Cathodes to Enhance Longevity of SiO/Graphite | NCM Full cells

**DOI:** 10.1002/advs.202518317

**Published:** 2025-11-26

**Authors:** Hyoyeong Kim, Chan Myeong Kim, Sangheum Jo, Seonghun Lee, Soon Ju Choi, Hyun Joo Park, Hyein Yu, Daesoo Kim, Kyungjun Kim, Tae Joo Shin, Sang‐Min Lee

**Affiliations:** ^1^ Department of Battery Engineering Graduate Institute of Ferrous & Eco Materials Technology (GIFT) Pohang University of Science and Technology (POSTECH) 77, Cheongam‐Ro Nam‐Gu Pohang 37673 Republic of Korea; ^2^ Graduate School of Semiconductor Materials and Devices Engineering Ulsan National Institute of Science and Technology (UNIST) 50 UNIST‐gil Ulsan 44919 Republic of Korea; ^3^ LG Energy Solution, Research Park 188, Munji‐ro Yuseong‐gu Daejeon 34122 Republic of Korea; ^4^ Department of Materials Science and Engineering Pohang University of Science and Technology (POSTECH) 77, Cheongam‐Ro Nam‐Gu Pohang 37673 Republic of Korea

**Keywords:** bimodal cathode design, cathode reaction heterogeneity, discharge regulation of SiO, parallel circuit modeling cell, SiO/graphite anode

## Abstract

High‐energy‐density lithium‐ion batteries are crucial for accelerating the widespread adoption of electric vehicles. Silicon monoxide/graphite (SiO/Gr) composite anodes have attracted considerable attention as promising candidates for increasing energy density. However, severe capacity degradation caused by the large volume changes of SiO during charge–discharge cycles remains a major obstacle to commercialization. One effective strategy to address this issue is to limit the charge/discharge operating voltage range (swing range) of the SiO anode. In this study, a cathode design composed of single‐crystalline and polycrystalline LiNi_0.8_Co_0.1_Mn_0.1_O_2_(NCM811) with a bimodal particle size distribution is proposed to effectively control the charge–discharge operating range of the SiO anode within a full‐cell. This design leverages the reaction heterogeneity of the cathode particles to induce an increase in overpotential at the end of discharge, effectively lowering the discharge endpoint potential of the anode. This design strategy enables stable cycling performance without compromising full‐cell energy density by selectively controlling the discharge depth of SiO in the SiO/Gr anode. The effectiveness of this design is validated through various electrochemical analyses and real‐time operando X‐ray Diffraction (XRD), demonstrating that it is an efficient strategy to enhance the long‐term cycle stability of SiO/Gr anodes without sacrificing energy density.

## Graphical abstract

A bimodal cathode composed of single‐ and polycrystalline NCM particles induces end‐of‐discharge overpotential rise via reaction heterogeneity, effectively regulating the depth of discharge of SiO in SiO/graphite anodes. This design suppresses mechanical degradation of SiO and enables long‐term cycling stability without compromising the energy density of full cells.

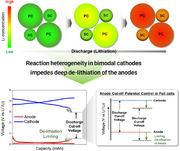

## 1 Introduction

Lithium‐ion batteries (LIBs) have firmly established themselves as the leading technology in the portable electronics market and are now a key driver of the transition from traditional internal combustion engine vehicles to electric vehicles (EVs).^[^ ^1^, ^2^, ^3^, ^4^, ^5^ ^]^ The continued growth of the EV industry requires next‐generation LIB technologies with higher energy density and stable long‐term cycle performance. Anode materials play a crucial role in determining battery energy density and cycle life, but graphite (Gr), the most widely used, is nearing its limits. Despite its excellent electrical conductivity and cycle stability, graphite's capacity is constrained by a theoretical limit of 372 mAh g^−1^, hindering further energy density improvements.^[^ ^6^, ^7^, ^8^ ^]^ To overcome the limitations, silicon (Si) and its oxides (SiO), with their high theoretical capacities, have emerged as promising alternative anode materials.^[^ ^9^, ^10^, ^11^, ^12^, ^13^, ^14^ ^]^ Among these, SiO offers greater cycling stability than pure Si, making it a suitable candidate for combining with graphite in SiO/Graphite (SiO/Gr) composite electrodes. This approach integrates the long‐term cycle stability of graphite with the high theoretical capacity of SiO (≈1500 mAh g^−1^), representing an effective strategy for boosting the energy density of lithium‐ion batteries.^[^ ^15^, ^16^, ^17^, ^18^ ^]^

**Figure 1:**
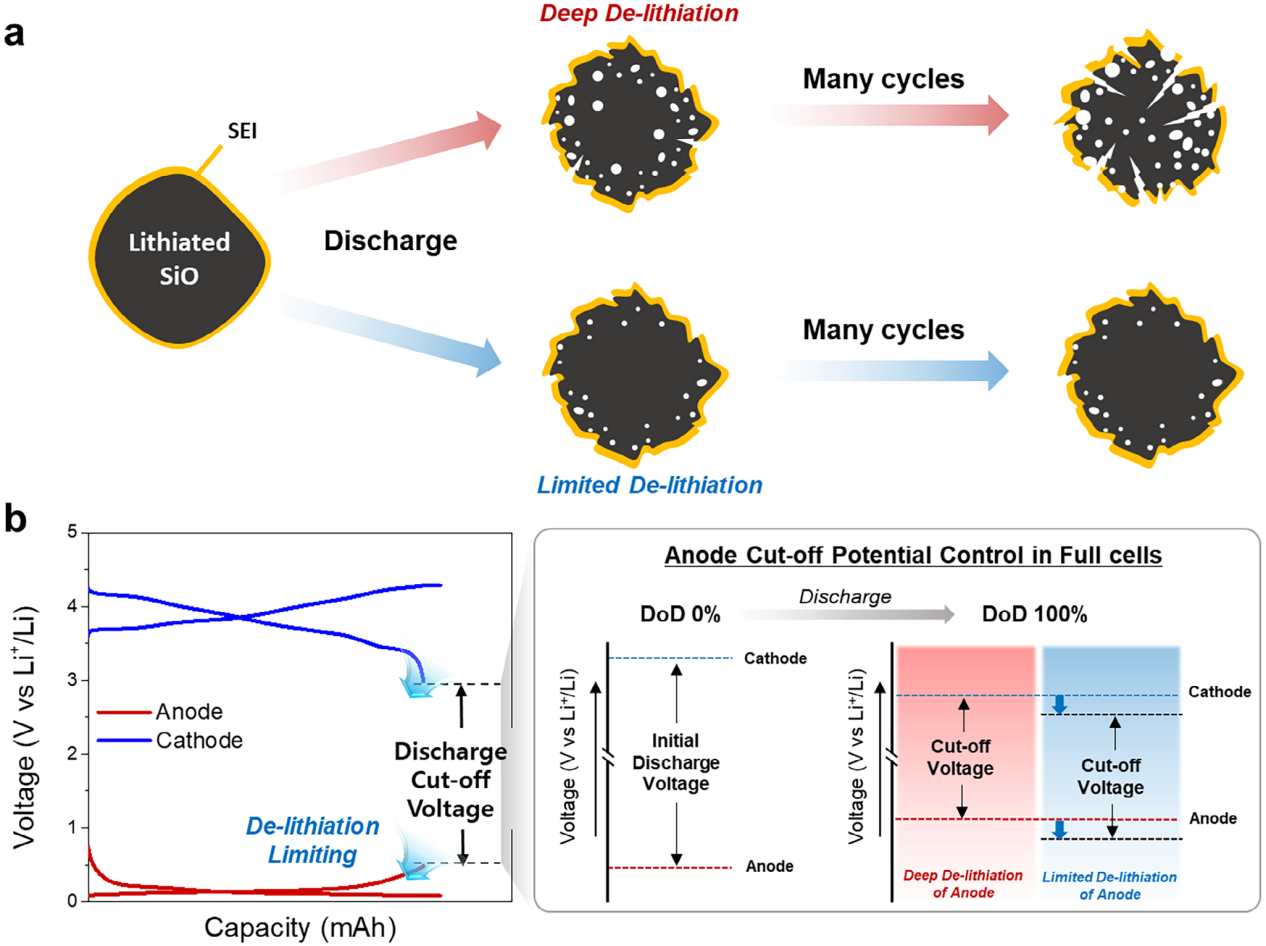
Schematic illustration of SiO degradation at varying discharge depths and anode discharge limiting method in full cells. a) Schematic illustration of the degradation process of SiO particles based on the Depth of Discharge (DoD). b) Each electrode potential profile of anode and cathode in a 3E‐full‐cell, and a schematic illustration for limiting DoD of the anode side at fixed discharge cut‐off voltage.

However, SiO undergoes significant lattice volume changes (≈200%) during lithiation and delithiation, which reduces the structural stability of the electrode and deteriorates the cycling performance. These challenges also affect SiO/Gr composite electrodes, necessitating improvements in long‐term cycle life. To address these challenges, extensive research has been conducted to enhance the cycle life of SiO/Gr composite electrodes.^[^ ^19^, ^20^, ^21^ ^]^ Among various approaches, reducing the depth of discharge (DoD) for SiO by limiting its delithiation range has been identified as a promising solution to suppress capacity fading. Lowering the discharge cut‐off voltage relative to Li^+^/Li effectively suppresses SiO delithiation, mitigates volume changes, and prevents particle pulverization, a key failure mode of silicon‐based anodes (**Figure** **1**a). This approach minimizes structural degradation in SiO particles during cycling, providing a straightforward and effective means to enhance cycle stability.^[^ ^22^, ^23^, ^24^, ^25^ ^]^

Recent approaches for controlling the delithiation depth of anodes in full cells have explored the use of over‐lithiated transition metal oxides, such as Li_2_NiO_2_ and Li_6_CoO_4_, as sacrificial cathode additives.^[^ ^26^, ^27^, ^28^, ^29^ ^]^ These materials exhibit exceptionally low coulombic efficiency due to their large irreversible capacity. During charging, they release excess lithium to compensate for the initial lithium loss, while during discharge, they only accept a small amount of lithium, thereby limiting the delithiation of the anode. This mechanism allows sacrificial cathodes to regulate the delithiation depth of SiO/Gr anodes and improve their electrochemical performance. However, their high sensitivity to moisture and structural instability in the delithiated state can adversely affect thermal safety and high‐temperature storage reliability.^[^ ^30^, ^31^ ^]^ Furthermore, after the initial activation, these materials become inactive components within the electrode, reducing the overall energy density of the cell.

In this study, a cell design strategy incorporating a cathode design with Bimodal (BM) particle size distribution is presented to regulate the depth of discharge (DoD) of SiO/Gr anodes as part of an approach to mitigate capacity degradation in full cells. This design implements DoD control of the anode in LIB full cells and effectively suppresses the capacity degradation of SiO/Gr anodes without causing energy density loss or other reliability issues (Figure 1b). The BM cathode employed in this study is designed to exploit inter‐particle reaction heterogeneity by tailoring distinct lithium transport kinetics of cathode particles with contrasting microstructural characteristics. Previous operando X‐ray diffraction (XRD) analyses of LiNi_1‐x‐y_Co_x_Mn_y_O_2_ (NCM) cathodes, typically regarded as single‐phase reaction materials, have shown phase separation during the charge/discharge process.^[^ ^32^, ^33^, ^34^, ^35^ ^]^ This phase separation is primarily attributed to inter‐particle reaction heterogeneity rather than intra‐particle reaction heterogeneity and has been mostly observed during charging. However, the application of a BM cathode design induces phase separation even during discharge, as demonstrated in XRD results.^[^ ^36^ ^]^ In the BM cathode system of this study, the single‐crystalline (SC, D_50_ 3.8 µm) and polycrystalline (PC, D_50_ 12.8 µm) NCM particles exhibit distinct reaction kinetics during discharge. PC particles react more actively in the early stages of discharge, while SC particles predominantly contribute to lithiation in the later stage. This inter‐particle reaction heterogeneity leads to a higher overpotential in the BM cathode compared to the Unimodal (UM) cathode, particularly at the end of discharge. At low current densities, the overpotential difference between BM and UM cathodes is minimal, but at higher currents, the BM cathode exhibits a significant increase in overpotential toward the end of discharge, leading to a pronounced voltage drop. This mechanism allows effective control over the DoD of SiO/Gr anodes, mitigating capacity degradation associated with repeated cycling.

The feasibility of the proposed cell design is validated through electrochemical analyses, including Galvanostatic Intermittent Titration Technique (GITT) and three‐electrode voltage monitoring. These measurements show that the BM cathode develops an increased overpotential near the end of discharge, enabling precise regulation of the SiO/Gr anode's discharge cut‐off voltage. A model cell, designed to isolate the reactions of individual active materials, clearly reveals inter‐particle reaction heterogeneities within the BM cathode. Synchrotron operando XRD further provides real‐time tracking of structural changes in both electrodes, verifying the design's effectiveness. The approach is demonstrated in pouch‐type full cells with SiO/Gr composite anodes and either UM (SC only) or BM cathodes. After 150 cycles, the SiO/Gr–BM cell retained 95.8% of its initial capacity, while the SiO/Gr–UM cell showed a significant capacity reduction to 80.8%, highlighting a clear difference in cycling performance. We propose that applying the BM cathode design, which combines single‐crystalline and polycrystalline LiNi_0.8_Co_0.1_Mn_0.1_O_2_ (NCM811) particles, can markedly improve the cycling performance of lithium‐ion batteries incorporating SiO/Gr composite anodes without compromising energy density or safety.

## 2 Results and Discussion

### 2.1 Electrochemical Delithiation Process of SiO/Gr Anodes in Lithium‐Ion Batteries

**Figure 2:**
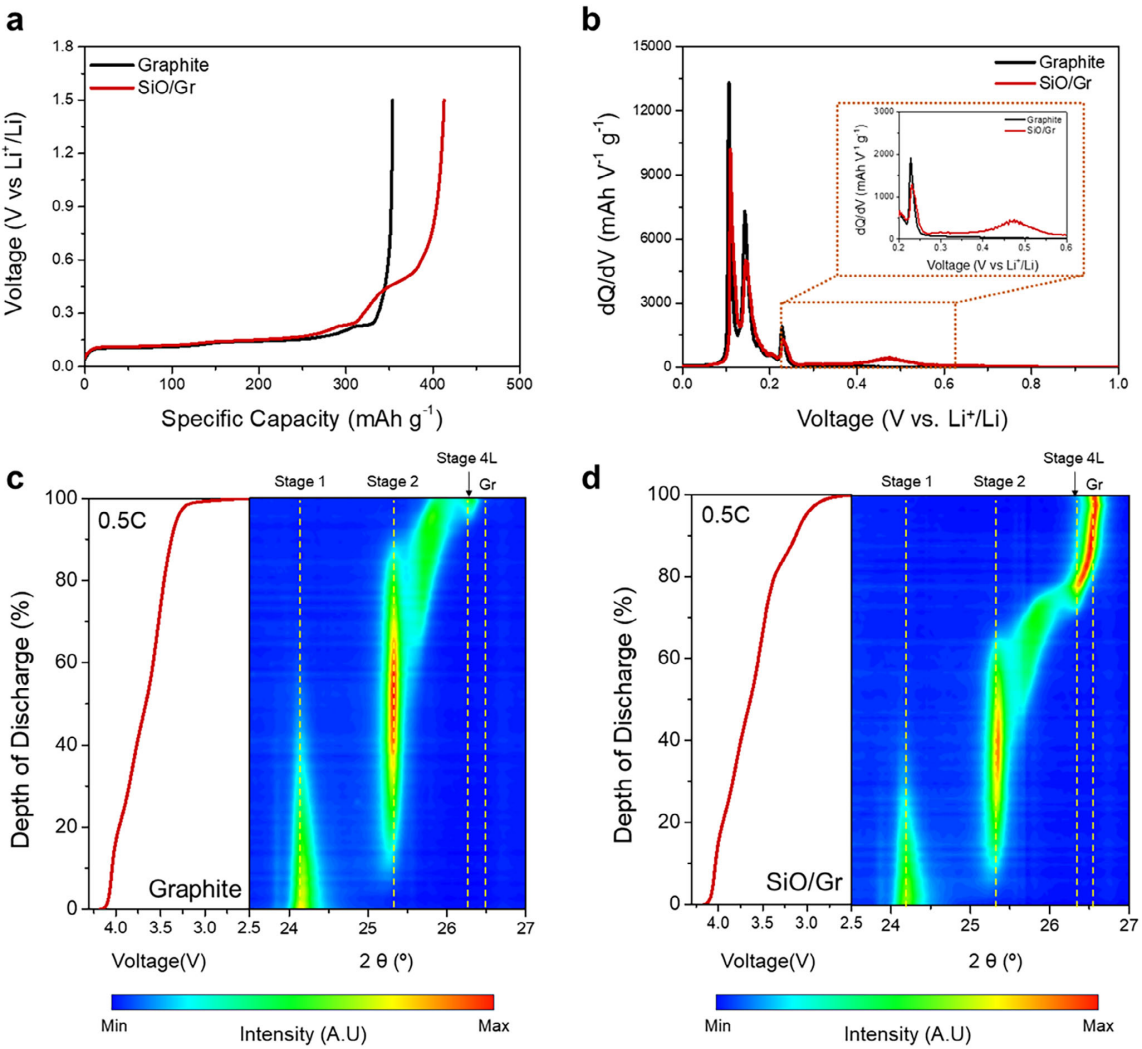
Comparison of delithiation processes between graphite and SiO/Gr anodes. a) Voltage profiles of graphite and SiO/Gr half cells during delithiation process at 0.1 C. b) Differential capacity (dQ/dV) plots of graphite and SiO/Gr half cells during delithiation process at 0.1 C. Operando XRD contour plots of graphite and corresponding voltage–time curves during 0.5 C discharge for c) Graphite | NCM811 and d) SiO/Gr | NCM811.

To compare the electrochemical delithiation behavior of graphite and SiO/Gr composite anodes, electrochemical charge–discharge tests are conducted using half cells with lithium metal as a counter electrode. **Figure** 2a presents the voltage profiles for the delithiation process of graphite and SiO/Gr half cells. As observed in the discharge curves, the primary delithiation reactions of the SiO and graphite occur at distinct potentials (V vs. Li⁺/Li).^[^ ^37^, ^38^ ^]^ In the voltage profile of the SiO/Gr anode, given the higher oxidation potential of SiO compared to graphite, it is expected that SiO delithiation primarily occurs during the later stages of the discharge process (Figure 2b).

To directly observe the crystallographic changes during the delithiation process of the SiO/Gr anode, operando XRD experiments are performed on full cells using graphite and SiO/Gr anodes. The resulting contour plots and voltage–time curves for the graphite anode and SiO/Gr anode are presented (Figure 2c,d). During lithiation/delithiation, the Si nanocrystal domains in SiO become amorphous, and no distinct Si diffraction peaks are observed.^[^ ^39^ ^]^ Therefore, the delithiation behavior of SiO is indirectly analyzed by monitoring the changes in the (002) diffraction peak of graphite, which exhibits distinct peak transitions during cycling. During discharge, the graphite electrode undergoes stepwise phase transitions as lithium is extracted. Operando XRD analysis revealed that, at the initial stage of discharge, LiC_6_ (Stage 1) transitions to LiC_12_ (Stage 2) as lithium is removed, and intermediate phases such as LiC_18_ or LiC_24_ are briefly maintained during further delithiation. By the end of discharge, most of the lithium is delithiated, returning the graphite to the LiC_72_ (Stage 4L) state (Figure 2c). In contrast, for the SiO/Gr electrode, the (002) peak associated with the LiC_72_ (Stage 4L) emerged at ≈80% DoD and exhibited minimal shifts until the end of discharge. This indicates that in the SiO/Gr electrode, delithiation of graphite is nearly complete before the later stage of discharge (DOD > 80%), with the reversible capacity primarily attributed to the delithiation of SiO. These findings indicate that, under the same cell‐level discharge cut‐off voltage, a cathode design strategy that induces a lower end‐of‐discharge potential can effectively limit the extent of SiO delithiation in full cells with SiO/Gr anodes, as illustrated in Figure 1b.

### 2.2 Bimodal Cathode‐Driven Control of SiO/Gr Anode Depth of Discharge

**Figure 3:**
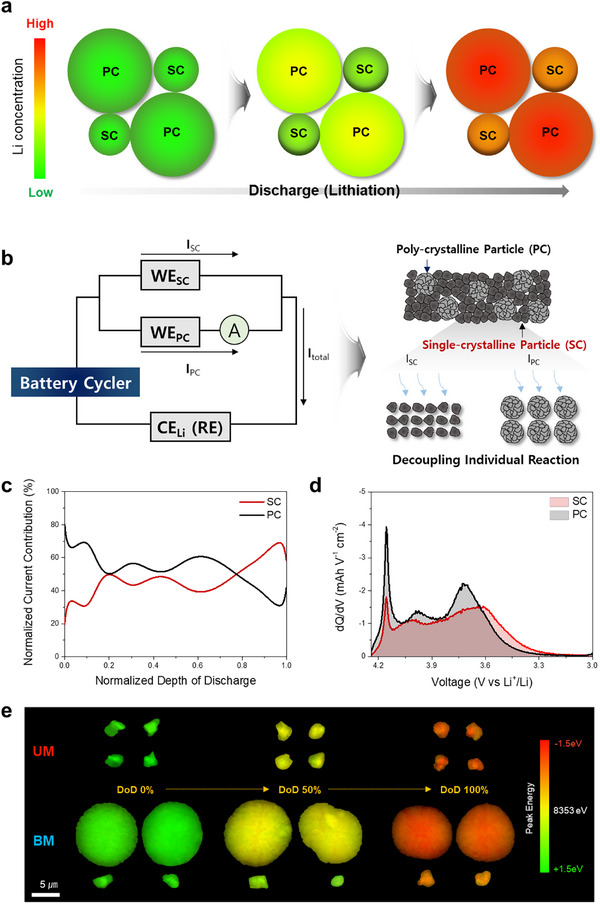
Inter‐particle reaction heterogeneity within the bimodal cathode during discharge. a) Schematic illustration of the lithium concentration variation of cathode particles during the discharge (lithiation) process of a bimodal cathode. b) Parallel Circuit Reaction Decoupling (PCRD) cell system to simulate the current distribution within the bimodal electrode during discharge. c) Normalized current contribution profiles of SC and PC cathodes during discharge, which is calculated using current values measured at 0.5 C from PCRD_BM cell. d) Differential capacity (dQ/dV) plots of SC and PC cathodes in PCRD_BM cell during discharge at 0.5 C. e) TXM‐XANES mapping for cathode particles in UM and BM cathode as a function of DoD.

In bimodal cathodes composed of NCM particles of different sizes, inter‐particle lithium‐concentration gradients may arise due to size‐dependent reaction kinetics during discharge, a phenomenon that manifests as phase separation observed in operando XRD.^[^ ^36^ ^]^ In this study, we leveraged the reaction rate disparities inherent to bimodal cathodes to modulate the reaction overpotential of the cathode during the late discharge stage. **Figure** **3**a illustrates the mechanism underlying the increase in reaction overpotential at the end of discharge in bimodal cathodes. The bimodal cathode is structured with a mixture of SC NCM811 and PC NCM811 particles. During discharge, differences in the reaction rates between SC and PC particles cause PC particles to reach specific lithium concentrations faster than SC particles.^[^ ^40^ ^]^ As a result, lithiation reactions are predominantly localized to SC particles toward the end of discharge. This leads to a concentrated current distribution on SC particles, imposing a high effective C‐rate, and consequently, a significant increase in reaction overpotential at the late discharge stage.

To measure the current distribution across SC and PC particles within the bimodal cathode, a Parallel Circuit Reaction Decoupling (PCRD) cell system is employed to simulate the bimodal electrode structure (Figure 3b). This system has been validated as an effective method for independently analyzing the charge/discharge characteristics of individual active materials in mixed‐material electrodes (e.g., SiO/Gr composite anodes or blended cathodes).^[^ ^41^, ^42^, ^43^, ^44^, ^45^ ^]^ In the bimodal electrode, SC and PC particles are electrically interconnected through direct physical contact and conductive carbon, resembling two single electrodes with distinct electrical resistances connected in parallel. In the PCRD cell system, the current flowing through the cell is expressed by Equation (1).

(1) $$I_{Total}=I_{SC}+I_{PC}$$

The total current applied to the circuit is distributed based on the resistance of each electrode, allowing the current through individual electrodes to be quantitatively analyzed. This enables real‐time differentiation of the reaction behavior between SC and PC particles. A cell assembled with SC and PC electrodes, each having identical areal capacities, is designated as PCRD_BM (Figure S1, Supporting Information). To evaluate the validity of the PCRD_BM cell, the charge–discharge voltage profile and dQ/dV plot obtained at a 0.1 C rate are compared with those of a conventional LIB coin cell (Conventional Cell_BM). The comparison revealed that the PCRD_BM cell exhibited nearly identical voltage profiles to the Conventional Cell_BM, with similar phase transition peaks characteristic of NCM811 observed in the dQ/dV plot (Figure S2, Supporting Information). Both systems showed phase transition peaks at ≈3.7 and 4.1 V, corresponding to the H1‐M and H2‐H3 transitions, respectively. These results confirm that the PCRD system can reliably simulate the electrochemical reaction behavior of NCM811, comparable to a conventional coin cell.

Changes in I_SC_ and I_PC_ as a function of lithiation time during discharge are illustrated (Figure S3, Supporting Information). The measured current values are normalized to I_Total_ and presented as normalized current distributions, with the variations in current distribution as a function of DoD shown in Figure 3c. In the early discharge region (DoD < 0.2), lithiation reactions are predominantly concentrated in the PC particles, resulting in a faster increase in lithium concentration in PC, compared to SC particles. In the late discharge region (DoD > 0.8), the lithiation activity in PC particles decreased, causing a shift in current concentration toward the SC particles. Consequently, more than 60% of I_Total_ is directed to SC particles during the later discharge stage, increasing the effective C‐rate applied to SC particles and raising the overpotential.

Figure 3d illustrates the differential capacity (dQ/dV) curves of SC and PC electrodes during the 0.5 C discharge process in the PCRD_BM cell. Consistent with the results in Figure 3C, PC particles exhibited major capacity contributions above ≈3.6V, whereas SC particles primarily contributed to lithiation reactions below 3.6V.

The effect of inter‐particle reaction heterogeneity on overpotential increase is evaluated using SC cathodes, PC cathodes, and BM cathodes comprising both SC and PC particles (Figure S4, Supporting Information). Galvanostatic Intermittent Titration Technique (GITT) measurements are conducted for each electrode using lithium metal as the counter electrode (Figure S5a, Supporting Information). The BM cathode exhibited a more pronounced increase in overpotential at the end of discharge compared to the SC or PC cathodes, with values of 143, 231, and 335 mV for SC, PC, and BM cathodes, respectively. Overpotential changes during discharge are visualized, clearly illustrating this trend (Figure S5b, Supporting Information). These results indicate that, in BM cathodes, the effective current density concentrates in SC particles toward the end of discharge, causing a sharp overpotential increase relative to cathodes composed exclusively of SC or PC particles.

**Figure 4:**
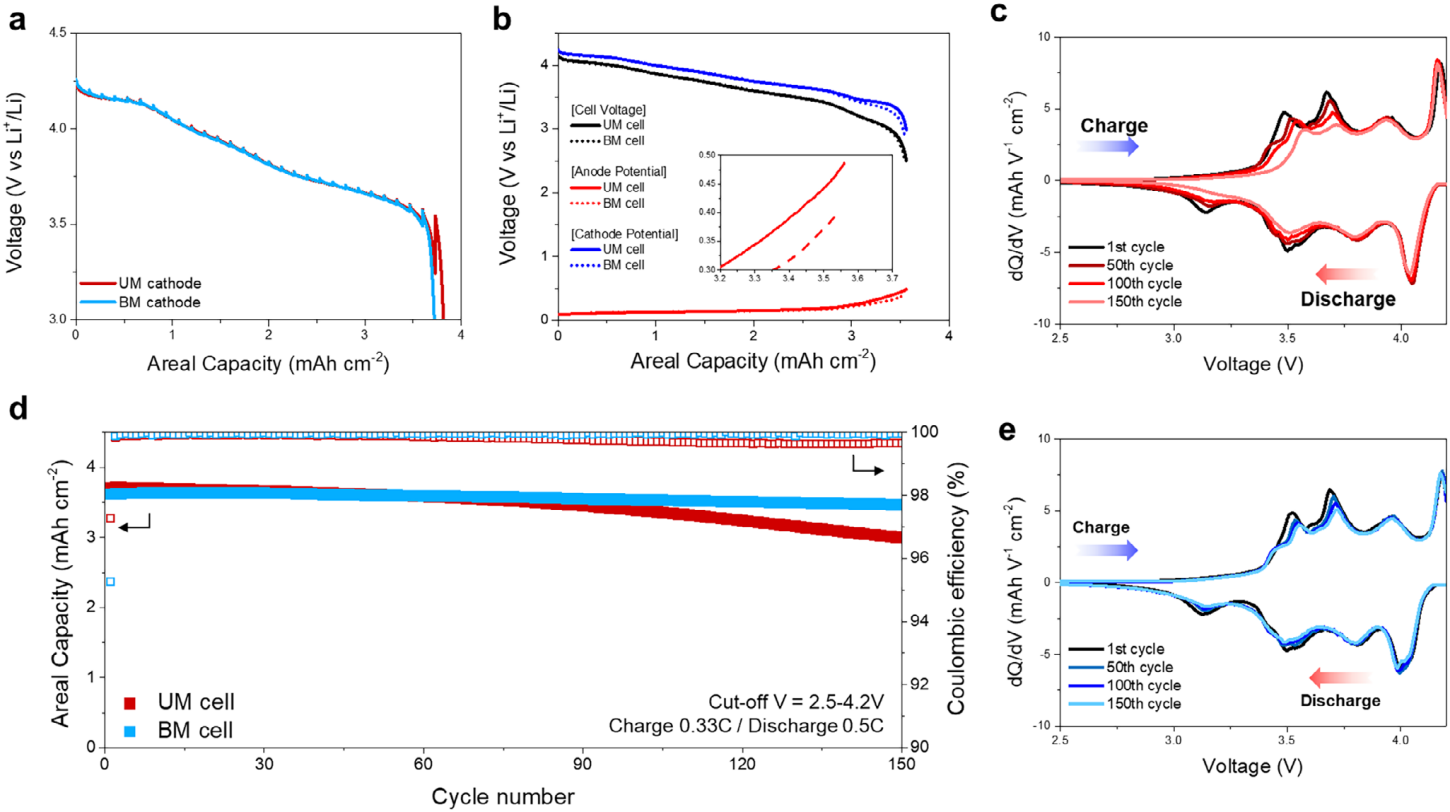
Cycling stability of SiO/Gr | NCM full cells. a) Galvanostatic Intermittent Titration Technique (GITT) voltage profiles for Li | NCM half cells with UM or BM cathode design during 0.5 C discharge. b) Voltage profiles of three‐electrode coin cells with SiO/Gr | UM and SiO/Gr | BM configurations during 0.5 C discharge. c) Differential capacity (dQ/dV) plots of UM cell d) Cycling performance of the UM and BM pouch cells, plotted as areal discharge capacity (mAh cm^−2^) e) Differential capacity (dQ/dV) plots of BM cell.

To verify the effect of BM cathode design on regulating the delithiation of SiO in SiO/Gr electrodes in a practical full cells, GITT and three‐electrode tests are performed using UM cathodes composed of only SC particles and BM cathodes containing a mixture of SC and PC particles (**Figure** **4**a,b). The areal capacity of both UM and BM cathodes was identically set to 4.05 mAh cm^−2^ (Figure S6, Supporting Information). The GITT voltage profiles during the 0.5 C discharge process showed a significant voltage drop near the end of discharge for the BM cathode compared to the UM cathode. As inferred from the PCRD experiments discussed earlier, the BM cathode experienced a pronounced increase in overpotential near the end of discharge, resulting in a discharge capacity that is 0.09 mAh cm^−2^ lower than that of the UM cathode.

The electrochemical lithiation behavior of SC and PC particles as a function of depth of discharge (DoD) is analyzed using transmission X‐ray microscopy coupled with X‐ray absorption near‐edge structure (TXM‐XANES) mapping (Figure 3e). Since Ni serves as the primary redox center during cycling in Ni‐rich layered oxides, variations in the Ni K‐edge peak positions among individual particles provide a sensitive indicator of inter‐particle reaction heterogeneity. In the UM cathode, the SC particles exhibited minimal differences in Ni oxidation states throughout discharge, maintaining a relatively homogeneous reaction behavior even at full discharge. By contrast, the BM cathode displayed pronounced inter‐particle differences between PC and SC particles. At DoD 0%, the Ni oxidation states are comparable across particle types. However, at DoD 50%, PC particles exhibited lower Ni oxidation states relative to SC particles, suggesting accelerated lithiation in PC particles during the early discharge stages. This trend is consistent with the earlier PCRD results, where lithiation preferentially occurred in PC particles at the onset of discharge. At DoD 100%, lithiation became increasingly concentrated in SC particles, and due to the elevated overpotential, lithiation terminated at a relatively lower lithium content. As shown in Figure S7 (Supporting Information), the mean difference in the Ni K‐edge peak positions between PC and SC particles is ≈0.5 eV at DoD 50% and remains ≈0.3 eV even at full discharge. In contrast, the UM cathode exhibits negligible peak position differences (< 0.1 eV) among particles throughout discharge, indicating highly homogeneous lithiation behavior with minimal inter‐particle reaction heterogeneity.

The influence of cathode overpotential behavior on SiO delithiation in the anode is evaluated using a three‐electrode full‐cell with an SiO/Gr anode, an NCM cathode (UM or BM), and a lithium reference electrode. The schematic of the three‐electrode configuration is shown in Figure S8 (Supporting Information), with experimental results in Figure 4b. Under 0.5 C discharge conditions, the potential of SiO/Gr anode at the end of discharge is 486 mV (vs. Li⁺/Li) with the UM cathode and 396 mV (vs. Li⁺/Li) with the BM cathode, indicating a ≈100 mV lower discharge cut‐off voltage with the BM cathode. This difference indicates that the high overpotential observed at the BM anode near the end of discharge substantially limits the SiO delithiation of the cathode. The high overpotential of the BM cathode is expected to mitigate particle crushing during shrinkage and suppress cyclic degradation by lowering the anode's cut‐off potential and restricting SiO delithiation.

A Pouch‐type full cell with a negative/positive (N/P) ratio of 1.05 is assembled by combining an SiO/Gr anode and an NCM cathode (UM or BM), followed by electrochemical analysis to evaluate its cycling performance. Before conducting long‐term cycling tests, charge–discharge experiments are performed at a low C‐rate (0.05 C) within a voltage range of 2.5–4.2 V. The full cells with UM cathodes (UM cell) and BM cathodes (BM cell) exhibited nearly identical voltage profiles, and their dQ/dV curves showed similar redox peaks throughout the charge–discharge process (Figure S9, Supporting Information). Unlike conventional strategies involving sacrificial cathode additives or increasing the N/P ratio, which lead to energy density loss, employing the BM cathode effectively demonstrated its capability to regulate SiO delithiation without compromising energy density (Table S1, Supporting Information).

Cycle performance is evaluated using a 0.33 C charge and 0.5 C discharge protocol within a voltage range of 2.5–4.2 V. In the first cycle, the Coulombic efficiency (CE) of the UM cell and BM cell is 97.28% and 95.26%, respectively, with the high overpotential at the end of discharge in the BM cathode likely contributing to a 2.02% lower CE compared to the UM cell (Figure S10, Supporting Information). The reproducibility of this observation is further validated by evaluating three additional cells of each type, as detailed in Table S2. During cycling, the capacity of the UM cell rapidly decreased to ≈80% of its initial capacity after 150 cycles, whereas the BM cell maintained ≈96% of its initial capacity, demonstrating more stable cycling performance (Figure 4d). The long‐term cycling performance plotted in terms of actual capacity and specific capacity (mAh g^−1^) is provided in Figure S11 (Supporting Information) for a more intuitive comparison. Furthermore, a comparison of CE values reflecting charge/discharge reversibility revealed that the CE of the UM cell gradually declined after ≈60 cycles, resulting in an average CE of 99.79% over the 2nd to 150th cycles. This decline indicates continuous irreversible lithium loss at the anode during cycling. In contrast, the BM cell maintained a higher average CE of 99.96% over the same cycle range.

To investigate capacity fading during long‐term cycling, dQ/dV plots are compared at the 1st, 50th, 100th and 150th cycles (Figure 4c,e). In SiO/Gr composite electrodes, lithiation begins with SiO at ≈0.4 V (vs. Li⁺/Li) and follows with graphite at ≈0.25 V during charging, while delithiation proceeds in reverse. Consequently, the dQ/dV peaks observed at the early charging stage and the end of discharge correspond to the reaction of SiO. In the UM cell, the lithiation (≈3.4 V) and delithiation (≈3.15 V) peaks of SiO are observed up to the 100th cycle, but disappear by the 150th cycle, indicating severe degradation from repeated deep delithiation. This suggests that SiO particles could no longer effectively participate in the reactions. In contrast, BM cells retained stable SiO peaks through 150 cycles, demonstrating that the high overpotential near the end of discharge in BM cathodes effectively regulates SiO delithiation, mitigating degradation and enhancing cycling performance.

### 2.3 Operando XRD Analysis of SiO/Gr Anodes: Elucidating Lithium Lithiation and Degradation Mechanisms in Long‐Term Cycling

**Figure 5:**
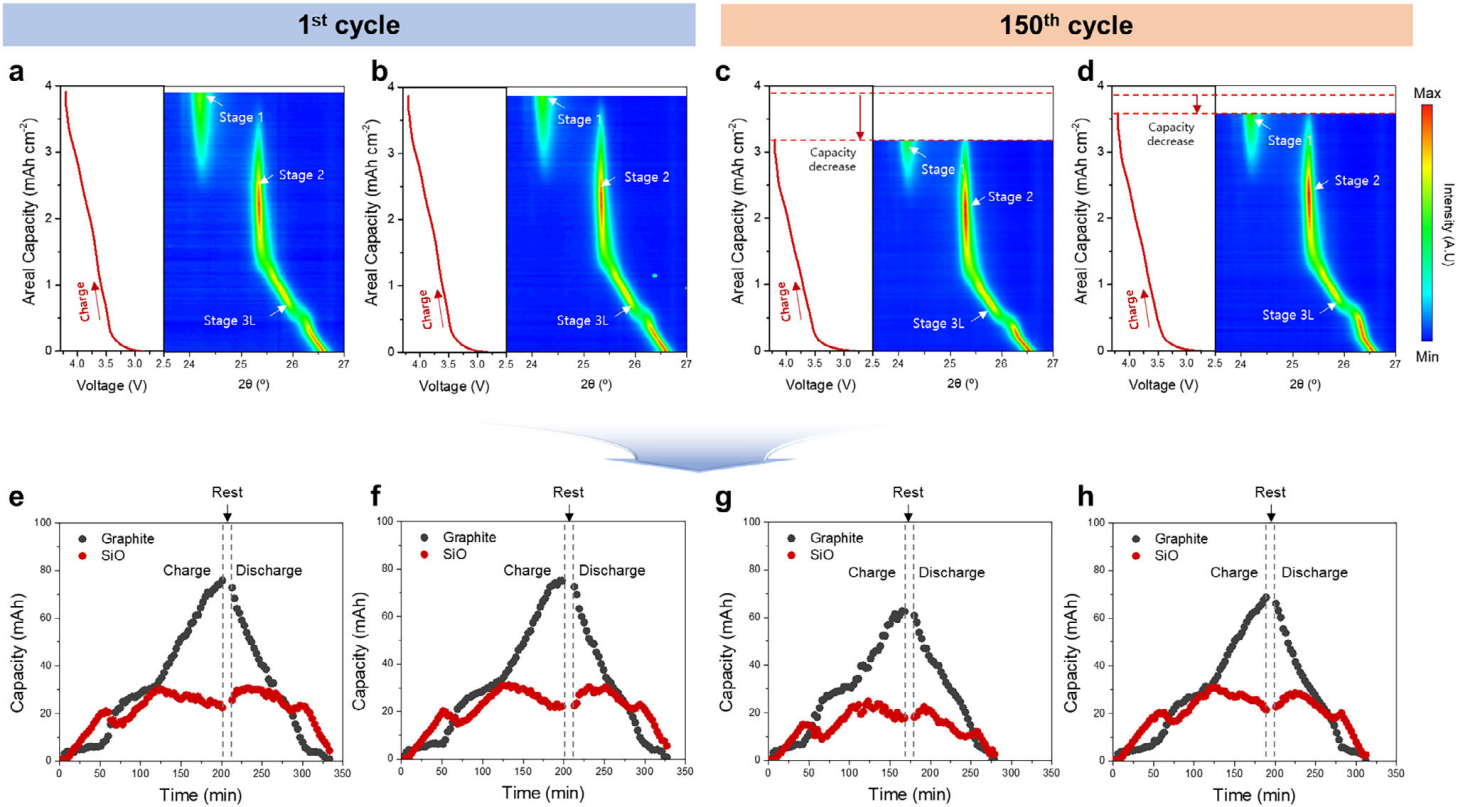
Operando XRD contour plots of the graphite and corresponding voltage–capacity curves at 0.33 C, presenting the first cycle for a) UM cell and b) BM cell, and the 150th cycle for c) UM cell and d) BM cell. Individual capacity profiles for the SiO and graphite at the first cycle for the e) UM cell and f) BM cell, and at the 150th cycle for the g) UM cell and h) BM cell.

Operando XRD analysis is performed to elucidate the degradation mechanisms of SiO within the anode during long‐term cycling. Results are compared with operando XRD profiles from full cells employing a SiO/Gr electrode, and Figure S12 (Supporting Information) presents the full charge–discharge XRD profiles of the UM and BM cells. To further analyze various phase changes at specific states of charge (SoC), the shifts in the graphite (002) diffraction peaks during the charging process are shown in **Figure** **5**a–d.

In the first cycle, the SiO/Gr composite electrodes exhibited delayed shifts in the graphite peak during the initial stages of charging, as the SiO particles underwent lithiation first. Compared to the graphite‐only electrode (Figures S13 and S14, Supporting Information), which displayed a Stage 3L phase transition at ≈0.4 mAh cm^−2^, the SiO/Gr electrodes showed the transition at ≈0.7 mAh cm^−2^. This trend is consistent in both the UM and BM cells (Figure 5a,b). However, significant differences are observed in the operando XRD profiles during the charging process of the 150th cycle between the UM and BM cells (Figure 5c,d). Specifically, in the UM cell, the transition to the Stage 3L phase occurred at a capacity that is ≈0.2 mAh cm^−2^ lower than that of the BM cell. The transition to Stage 3L occurred earlier during the initial charge with a charge difference of ≈0.2 mAh cm^−2^ compared to the BM cells. This shift suggests a reduction in the contribution of SiO lithiation during the initial charging stages, indicating degradation of the SiO anode in the UM cell.

Based on the XRD data obtained from full cells with graphite anodes, the variations in lithium content in SiO and graphite during the charge/discharge process of the full‐cell with a SiO/graphite composite cathode are indirectly estimated. The operando XRD data, combined with real‐time charge–discharge capacity data, are used to create a matrix polynomial model (Figures S15 and S16, Supporting Information) for calculating the contribution of each active material to the overall capacity. Figure 5e–h illustrates the lithium content in graphite and SiO within the SiO/Gr composite anode during cycling, as determined using this approach. The capacity profiles of individual active materials during the first cycle for UM and BM cells are presented in Figure 5e,f. Both cells exhibit a similar trend, where lithiation primarily occurs in SiO during the initial charging stages, while delithiation mainly proceeds from SiO in the later stages of discharge. Abrupt discontinuities in the individual capacity of SiO and graphite are observed, attributed to phase transitions in Li_x_Si that induce sudden potential shifts.^[^ ^21^, ^46^ ^]^ However, as shown in Figure 5g,h, the individual capacity profiles at the 150th cycle display notable differences between the two cells. In the UM cell, the capacity contribution of SiO, which initially dominated the initial charging and late discharging processes, is significantly reduced. The maximum capacity of SiO decreased by ≈7 mAh compared to the first cycle (30.5 mAh → 23.4 mAh). Additionally, irreversible lithium loss within the cell leads to a decrease in the capacity attributed to the lithiation of graphite. In contrast, the BM cell maintains a capacity contribution from SiO comparable to that in the first cycle, even at the 150th cycle. This suggests that the application of the BM cathode, which induces higher overpotential at the late discharge stage, effectively regulates SiO delithiation, thereby mitigating the degradation of SiO particles.

Additionally, the observation of diffraction peaks in the NCM cathode confirmed the trends identified in earlier electrochemical analyses. During the early stage of discharge, the NCM (003) diffraction peak splits (Figure S17, Supporting Information). Moreover, the separation of the (104) diffraction peak at both the beginning and end of discharge indicates inter‐particle reaction heterogeneity within the bimodal cathode (Figure S18, Supporting Information). These results suggest that differences in reaction kinetics between single‐crystalline and polycrystalline NCM particles lead to uneven lithium‐ion distribution.

### 2.4 Identifying the Main Causes of Capacity Decrease

**Figure 6:**
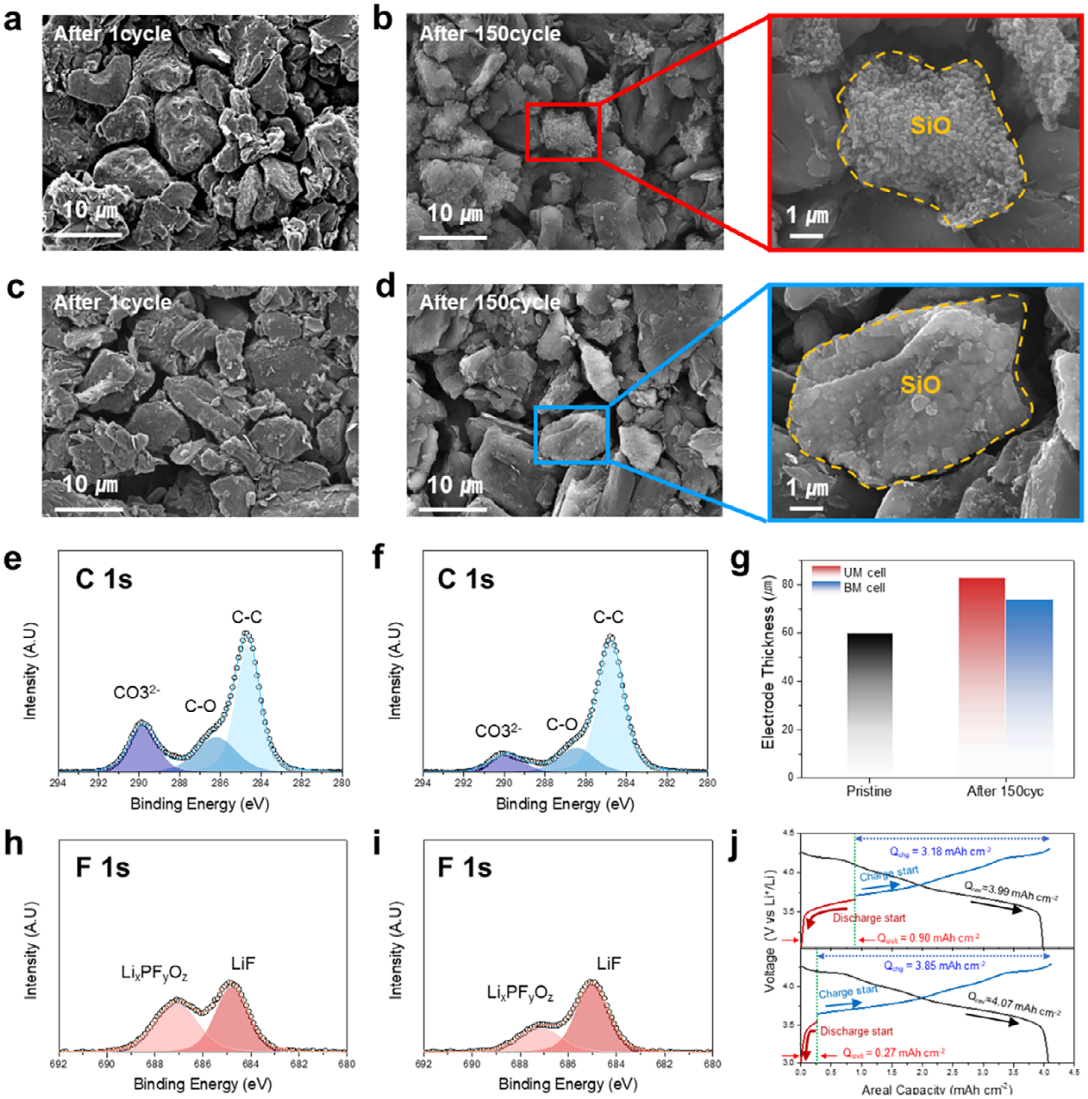
Post‐mortem analysis for SiO/Gr anodes. Top‐view SEM images of SiO/Gr anodes: UM cell after the a) one cycle and b) 150 cycles, and BM cell after the c) one cycle and d) 150 cycles. XPS C 1s spectra of SiO/Gr anodes after 150 cycles for e) UM cell and f) BM cell. g) Measured thickness of the SiO/Gr anodes before the cycle and after the 150th cycle. XPS F 1s spectra of the SiO/Gr anodes after 150 cycles for h) UM cell and i) BM cell. j) Voltage profiles of the reassembled coin cells with (top) UM cathode and (bottom) BM cathode, collected from pouch cells after 150 cycles.

The extent of active material degradation is assessed through comparative SEM imaging of SiO/Gr electrode surfaces collected from pouch full cells after 150 cycles. After only one cycle, both the UM and BM cells exhibited undamaged surfaces of SiO and graphite particles (**Figure** **6**a,c). However, after the 150 cycles, the SiO particles in the UM cell showed significantly roughened and porous structures due to continuous volume changes (Figure 6b). This degradation is attributed to surface cracking and structural damage caused by repetitive expansion and excessive contraction. In contrast, the BM cell maintained an overall smooth and stable SiO particle surface, suggesting that the use of the BM cathode effectively reduces the discharge depth of SiO in the anode and mitigates the resulting mechanical degradation of SiO particles (Figure 6d).

Compositional changes in the SEI layer on SiO particles induced by chemo‐mechanical degradation are analyzed by X‐ray photoelectron spectroscopy (XPS), comparing the chemical composition of SiO/Gr anode surfaces collected from pouch cells after the 150th cycle (Figure 6e,f,h,i). All XPS spectra were calibrated using the C 1s peak at 284.6 eV attributed to C─C bond as a reference. XPS spectra measured after the first cycle are presented (Figure S19, Supporting Information). The C 1s spectra of the SEI films on each SiO/Gr composite anode surface reveal no appreciable difference in the relative intensities of the C─C (284.6 eV) and C─O (286.3 eV) peaks (Figure 6e,f). However, the carbonate species (‐CO_3_, 289.7 eV), decomposition products of organic electrolytes, exhibited higher intensity in cells with UM cathodes.^[^ ^47^, ^48^, ^49^, ^50^ ^]^ Additionally, the F 1s spectra revealed that the decomposition product of the lithium salt (LiPF_6_), identified as Li_x_PF_y_O_z_ (687.5 eV), is also more pronounced in the UM cathode cells (Figure 6h). These findings suggest that the repeated expansion and contraction of SiO particles in the UM cathode cells disrupted the SEI layer, exposing fresh surfaces and accelerating electrolyte decomposition. The additional growth of the SEI layer can lead to the loss of active lithium within the cell, accelerating battery capacity degradation.^[^ ^45^ ^]^

To further examine the impact of cycling on the structural integrity of the SiO/Gr anode, the electrode thickness changes before and after cycling were observed (Figure 6g). The initial thickness of the SiO/Gr composite anodes is 60 µm. After 150 cycles, the UM cell exhibited a notable thickness increase to 83 µm, representing an expansion of ≈38.3%. In contrast, the BM cell exhibited a markedly lower expansion rate of 23.3%, with the anode maintaining a thickness of 74 µm (Figure S20, Supporting Information). The reduced SiO/Gr anode expansion in the BM cell can be attributed to the restricted delithiation of the SiO cathode by the BM cathode, which exhibits a higher overpotential at the end of discharge. Since delithiation from the SiO anode is controlled at the end of discharge, the pulverization of SiO particles is suppressed, thereby limiting side reactions with the electrolyte and ensuring uniform lithium‐ion charging. This is expected to provide a relative advantage in reducing electrode expansion during charging. These findings underscore the effectiveness of the BM cathode in enhancing the long‐term cycling stability of SiO/Gr composite anodes while preserving their structural integrity.

The contribution of the cathode to capacity fade in full cells is evaluated by collecting UM and BM cathodes after 150 cycles and reassembling them into half cells with Li metal as the counter electrode. Prior to disassembly, all cells underwent an additional discharge step (CC discharged at 0.5 C, followed by CV for 10 h at 2.5V) to ensure that any active residual lithium remaining in the anode is considered negligible. The voltage profiles for the additional‐discharge process, shown in Figure S21 (Supporting Information), reveal that restricting SiO delithiation in the BM cell retains a greater amount of electrochemically active residual lithium in the anode for up to 50 cycles. However, after 150 cycles, the cell resistance in the UM cell increases because of the accelerated degradation of SiO particles, which eventually prevents full delithiation even at a 0.5 C discharge rate, leaving more lithium in the SiO/Gr anode. After 150 cycles in the pouch cells, the collected UM and BM cathodes were reassembled into coin‐type half cells for two distinct purposes. For each configuration, one half‐cell is subjected to charging first, while the other is subjected to discharging first, to evaluate their respective capacities. The capacity measured during the initial charge is defined as Q_chg_, and the capacity measured during initial discharge is referred to as Q_shift_. These metrics are influenced by the irreversibly consumed lithium in the anode, which directly impacts the amount of lithium returned to the cathode structure. The subsequent discharge capacity following the initial charge is defined as Q_rev_, with Q_rev_ ‐ Q_shift_ closely matching the full‐cell capacity. Voltage profiles at 0.05 C for half cells employing UM and BM cathodes, collected from full cells disassembled after the formation process, are shown in Figure S22 (Supporting Information). Both cathodes exhibited almost identical Q_rev_, and the irreversible lithium loss in the anode, represented by Q_shift_, accounted for 3.89% (0.16 mAh cm^−2^) and 3.40% (0.14 mAh cm^−2^) of Q_rev_ for the UM and BM cathodes, respectively. However, after 150 cycles, significant differences in Q_shift_ were observed between the UM and BM cathodes (Figure 6j). The Q_rev_ of the UM cathode is only 0.08 mAh cm^−2^ lower than that of the BM cathode, a negligible difference relative to the total capacity. In contrast, the Q_shift_ in the UM cathode increased significantly to 0.90 mAh cm^−2^, compared to 0.27 mAh cm^−2^ in the BM cathode, corresponding to 21.90% and 6.55% of their initial reversible capacity, respectively. To further validate the above half‐cell test results, the (003) diffraction peak positions of the cathodes at 0% SoC were analyzed for the 1st and 150th cycle (Figure S23, Supporting Information). The UM cathode exhibited a shift to lower angles with increasing cycling, suggesting incomplete lithium reinsertion into the cathode structure even after the full‐cell is completely discharged. Additionally, SiO/Gr anodes collected after the 150th cycle exhibited a pronounced reduction in SiO redox activity in the UM cell compared to the BM cell. While the BM cell retained distinct (de)lithiation peaks of SiO in the 0.2–0.5 V (vs. Li⁺/Li) range, these peaks were significantly weakened in the UM cell, indicating substantial degradation of SiO electrochemical activity (Figure S24, Supporting Information). These findings indicate that capacity fading in UM full cells is primarily driven by irreversible lithium loss in the anode. In contrast, the BM cathode effectively limits deep delithiation of SiO particles, thereby mitigating structural deterioration. Notably, the capacity loss of the cathodes themselves is negligible compared to the influence of the anode in all tested full cells.

Nyquist plots obtained after the 50th and 150th cycles for both UM and BM cells, revealing a substantial increase in impedance for the UM cell (Figure S25, Supporting Information). To gain a more comprehensive understanding of impedance evolution, a distribution of relaxation times (DRT) analysis is conducted to deconvolute the impedance spectra.^[^ ^51^ ^]^ The DRT function (γ(τ)) is plotted against relaxation time (τ), enabling clear differentiation of reaction processes within the impedance data (Figure S26, Supporting Information). Three characteristic peaks were identified, each corresponding to a distinct electrochemical process. Peaks observed within the range of 10^−4^–10^−2^ s are associated with resistance from the SEI and CEI layer (R_SEI+CEI_), those within 10^−1^ to 1 s correspond to charge transfer resistance (R_ct_), and the peak appearing at τ > 1 s is related to Li^+^ diffusion resistance (R_Diff_).^[^ ^52^, ^53^, ^54^, ^55^ ^]^ Notably, compared to the BM cell, the UM cell exhibited a significant increase in R_SEI+CEI_ and R_ct_ at the 150th cycle. Based on the previous observations, this increase in resistance appears to be primarily driven by the degradation of SiO particles during cycling.

Cycling performance was evaluated under various current densities using pouch cells with electrolyte amounts close to commercial levels and coin cells with excess electrolyte (Figure S27, Supporting Information). In Si‐containing cells, particle fracture and progressive SEI growth accelerate electrolyte depletion, rendering lean‐electrolyte operation prone to rapid capacity fading.^[^ ^56^, ^57^, ^58^ ^]^ In pouch‐cell testing (E/C ratio = 2.0 µL mAh^−1^), SiO shrinkage during deep discharge likely contributes to electrolyte depletion, leading to pronounced differences in cycle life, as BM cells outperform UM cells (Figure S27a,b, Supporting Information). By contrast, coin‐cell testing (E/C ratio = 30.0 µL mAh^−1^) exhibits minimal differences owing to the electrolyte‐rich environment (Figure S27c,d, Supporting Information). These findings underscore that leveraging cathode reaction heterogeneity to control the SiO depth of discharge is an effective approach to extending cell lifespan under practical electrolyte constraints.

## 3 Conclusion

To address the capacity fade issue during the cycling of the SiO/Gr composite anode, this study proposes a strategy to control the depth of discharge (DoD) of SiO particles through the introduction of a bimodal cathode design. The distinct difference in reaction rates between the two types of cathode particles (SC and PC) in the bimodal electrode leads to a rapid increase in overpotential toward the end of discharge, effectively limiting the DoD of SiO/Gr anode in the full‐cell. Systematic electrochemical analyses and operando XRD diffraction patterns demonstrate that this design mitigates the pulverization of SiO particles and enhances the structural stability of the electrode. As a result, it achieves superior reversible capacity retention (95.8% after 150 cycles) compared to a full‐cell using a conventional unimodal design (80.8% after 150 cycles). Notably, this approach offers a strategy to enhance long‐term cycling stability without sacrificing energy density, in contrast to conventional sacrificial cathode design. This establishes the bimodal cathode design as a practical and scalable solution for extending the cycle life of high‐energy density batteries using SiO/Gr composite anode.

## 4 Experimental Section

### Preparation of Electrodes

Single‐crystal (SC), polycrystalline (PC), and bimodal (BM, a mixture of SC and PC) NCM811(Theoretical capacity = 200 mAh g^−1^) cathodes were fabricated to compare their electrochemical charge–discharge behaviors. The active material, Super P, and PVDF binder were mixed in a weight ratio of 90:5:5 in a 1‐methyl‐2‐pyrrolidinone (NMP) solvent to prepare a homogeneous slurry using a thinky mixer. The resulting slurry was coated onto Al foil (15 µm) using a doctor blade and dried in a vacuum oven at 100 °C for 10 h. The electrodes were then punched into 12 mm diameter disks. To minimize transport effects from the electrolyte, electrodes with a low active mass loading (≈ 5 mg cm^−2^) were prepared and used in the experiments.

The industrial‐grade graphite, SiO/Gr composite (Graphite: SiO = 94:6 wt.%), and NCM811 (UM and BM) electrodes used for cycle performance evaluation and operando XRD analysis were supplied by LG Energy Solution, Ltd. The anodes were composed of 95.4 wt.% active material (Graphite or SiO/Gr) and 4.6 wt.% inactive components (conductive additive and binder). The cathodes consisted of 97.2 wt.% active material (NCM) and 2.8 wt.% inactive components. The cathodes were designed with an areal capacity of ≈ 4.05 mAh cm^−2^, while the mass loading of the anodes was adjusted to achieve an N/P ratio of 1.05. The pouch cells comprised one double‐side‐coated anode (dimensions of 31 × 42 mm) and one single‐side‐coated cathode (dimensions of 30 × 41 mm), separated by a polyethylene porous film. A total of 200 µL of electrolyte was injected into each pouch cell. Four types of pouch cells were assembled for performance evaluation by combining the two types of anodes (Graphite and SiO/Gr) with the two types of cathodes (UM and BM) under identical conditions.

### Electrochemical Evaluation

CR2032 coin‐type cells were assembled in an Ar‐filled glovebox (KOREA KIYON, H2O < 0.1 ppm, O2 < 0.1 ppm). Polyethylene membrane (16 µm) was used as a separator, and the electrolyte consisting of 1.3 m LiPF_6_ in ethylene carbonate/diethyl carbonate (EC: EMC: DMC = 3:5:2 vol.%) with 5wt.% fluoroethylene carbonate (FEC). All pouch cells were positioned between two aluminum plates and tightened with a torque of 4 kgf • cm. Electrochemical charge–discharge tests were conducted using a battery cycler (WBCS3000, Wonatech Ltd.). The current density for the full cells was calculated based on an areal capacity of ≈4.05 mAh cm^−2^. The cells were charged to 4.2 V at a 0.33C rate, followed by constant voltage (CV) charging until the current decreased below 0.05 C. After a 10 min rest period, the cells were discharged to 2.5 V at a constant current corresponding to a 0.5 C rate.

To analyze the individual electrochemical behavior of the cathode and anode, three‐electrode coin cells were assembled. The reference electrode was prepared by attaching lithium foil to the end of a stainless steel (SUS, 10 µm) foil strip, with the SUS foil extending outside the coin cell and positioned along the cell's edge. Kapton tape and additional separators were used to prevent contact between the reference electrode and the cathode or anode.

The reaction overpotential of the cathode was compared using the Galvanostatic Intermittent Titration Technique (GITT) analysis. GITT measurements were conducted on cathode half cells after the formation process was completed. The protocol involved applying a 0.5 C constant current discharge for 4 min, followed by a 2 h rest period, repeated within the voltage range of 3.0–4.3 V (vs Li^+^/Li)

Electrochemical Impedance Spectroscopy (EIS) measurements were performed using a potentiostat (VSP‐300, BioLogic) with a 5 mV amplitude over a frequency range of 100 kHz–10 mHz. Analysis of distribution relaxation time (DRT) from EIS spectra was conducted by Matlab 2022 with a toolbox of DRT‐TOOLS developed by the research group of processor Francesco Ciuccil.

The Parallel circuit reaction decoupling (PCRD) cell system was constructed to decouple and analyze the reactions of individual active materials within composite electrodes. The lithium metal was placed between the two working electrodes, serving as both the reference and counter electrode. Electrical contact with the lithium metal was established using SUS foil, which was wrapped with Kapton tape to prevent short circuits. In the PCRD cell, the lithium metal counter electrode was centrally positioned, with the SC electrode (WESC) and PC electrode (WEPC) placed on opposite sides as working electrodes. These two working electrodes were connected in a parallel circuit. The voltage and applied current of the PCRD cell were monitored using a Battery Cycler (Wonatech, WBCS 3000 s), while the current distributed to each electrode was measured using a digital multimeter.

### Transmission X‐Ray‐Microscopy (TXM)

TXM‐XANES measurements were performed using full‐field transmission X‐ray nanoimaging at the 7 C beamline of the Pohang Light Source‐II (PLS‐II). Single‐pixel X‐ray absorption near‐edge structure (XANES) spectra were obtained with a spatial resolution of ≈30 nm. Images were recorded across the Ni K‐edge energy range of 8323–8403 eV, with an exposure time of 3.5 s per frame. Image alignment and spectral analysis were performed using the TXM Wizard software package.^[^ ^59^ ^]^ The samples for TXM analysis at specific DoD were prepared by promptly removing the electrodes from the cells within 2–3 min after current interruption and immediately rinsing them with diethyl carbonate (DMC) to prevent lithium exchange between particles.

### Operando X‐Ray Diffraction (XRD)

To observe real‐time structural changes during charge and discharge, operando XRD analysis was conducted at the beamline 6D at PLS‐II. The pouch cell under investigation was placed between two aluminum plates, with a small hole in the center of one plate to allow X‐ray beam passage. The beam energy and wavelength were 18.9 keV and 0.65297 Å, respectively. The diffraction patterns were recorded in transmission mode every 1 min and 30 s using a 2D CCD detector (Rayonix MX225‐HS, Rayonix). The recorded data were converted to match the conventional X‐ray beam source (Cu Kα, λ = 1.5406 Å) for ease of comparison.

### Cell Disassembly

For post‐mortem analysis, cycled Pouch cells were disassembled to retrieve the electrodes. Prior to disassembly, all cells underwent CC discharge at a current density of 0.05 C, followed by CV discharge at 2.5 V to minimize active residual lithium in the anode. The fully discharged pouch cells were transferred to an Ar‐filled glove box, where the electrodes were separated and rinsed with dimethyl carbonate (DMC). The rinsed electrodes were dried under vacuum at room‐temperature conditions for 1 h. For the half‐cell reassembly experiments, the dried electrodes were punched into 10 mm diameter discs for use.

### Characterization

Top‐view and cross‐sectional scanning electron microscopy (SEM) images were obtained using a field emission scanning electron microscope (FE‐SEM, JSM‐7800F, JEOL Ltd.), enabling detailed morphological characterization and the analysis of elemental distribution in the electrodes through energy‐dispersive spectroscopy (EDS) mapping. XPS analysis (Nexsa G2, Thermo Fisher) was performed using a monochromatic Al Kα source (15 kV) to investigate the chemical composition and surface states of the electrodes. Each spectrum was acquired with a pass energy of 50.0 eV and a step size of 0.1 eV. All spectra were calibrated using the C 1s C─C binding energy peak at 284.8 eV.

## Conflict of Interest

The authors declare no conflict of interest.

## Supporting information



Supporting Information

## Data Availability

The data that support the findings of this study are available from the corresponding author upon reasonable request.
