# Supplementary material for: Leveraging Reaction Heterogeneity in Bimodal Cathodes to Enhance Longevity of SiO/Graphite | NCM Full cells
Source: Adv Sci (Weinh). 2025 Nov 26;13(8):e18317. doi: 10.1002/advs.202518317 (PMC12884803; doi:10.1002/advs.202518317)
Supplement: Supplementary file 1 — Supporting Information [file ADVS-13-e18317-s001.docx]

Supporting Information

Leveraging Reaction Heterogeneity in Bimodal Cathodes to Enhance Longevity of SiO/Graphite | NCM Full cells

Hyoyeong Kim, Chan Myeong Kim, Sangheum Jo, Seonghun Lee, Soon Ju Choi, Hyun Joo Park, Hyein Yu, Daesoo Kim, Kyungjun Kim, Tae Joo Shin, Sang-Min Lee*


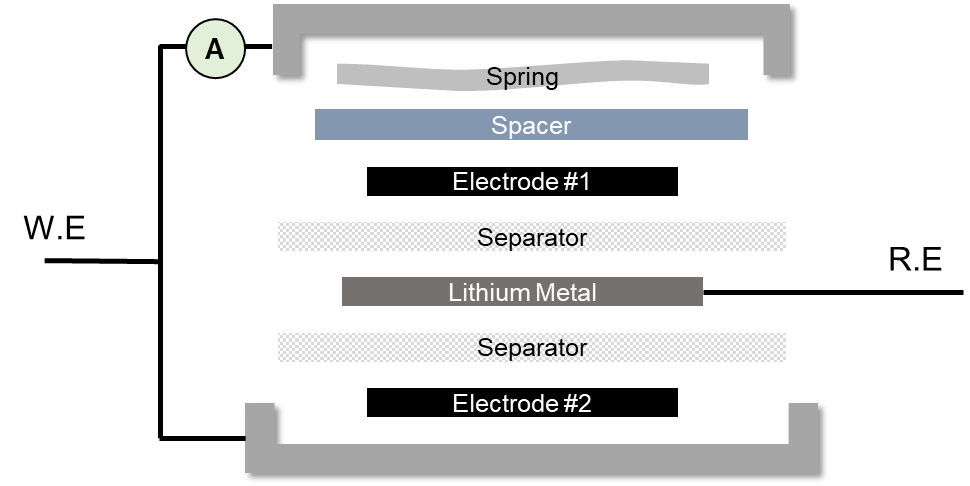


**Figure S1.** Schematic illustration of the PCRD cell configuration.


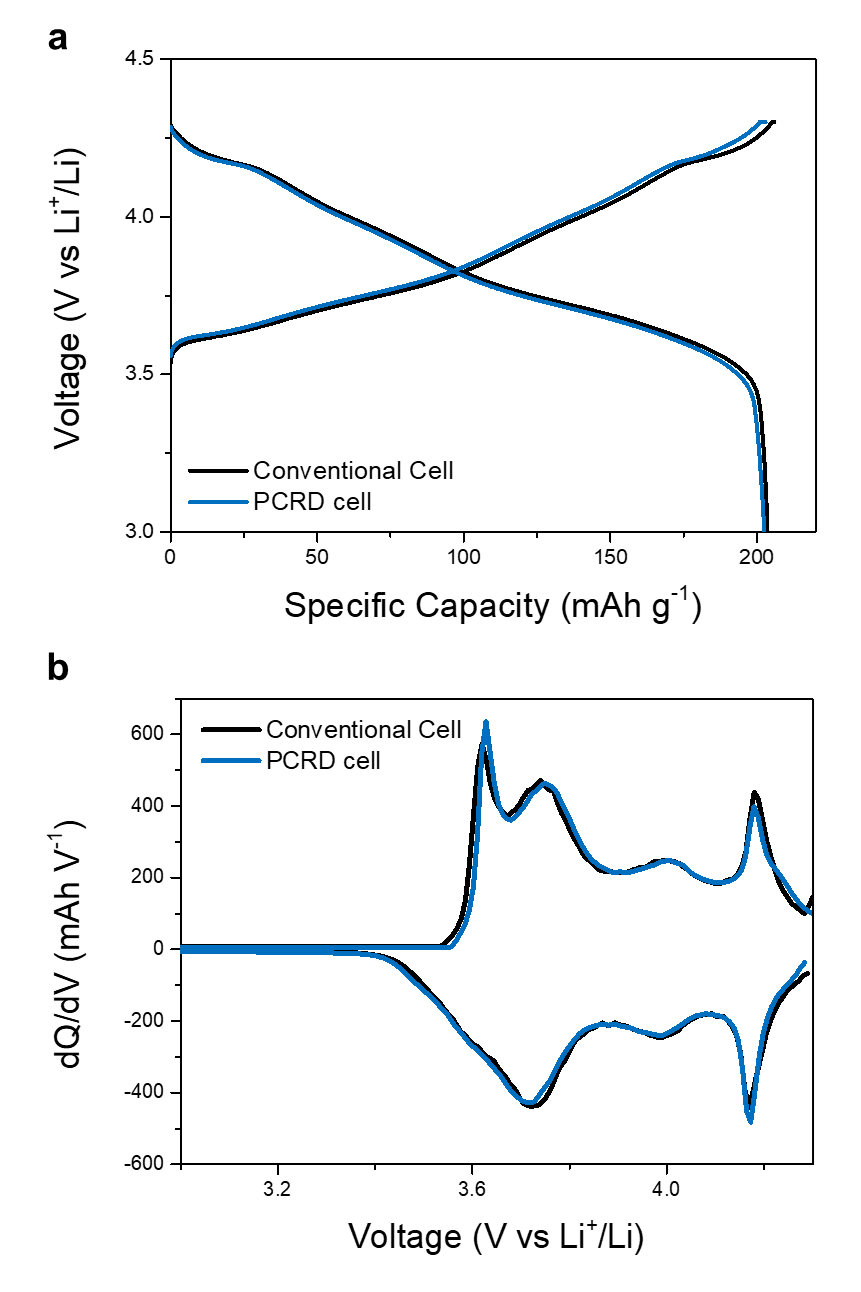


**Figure S2.** Validation of the PCRD Cell system. a) Charge-discharge voltage profiles of conventional cathode half-cell and PCRD cell. b) Differential Capacity(dQ/dV) plots of conventional cathode half-cell and PCRD cell


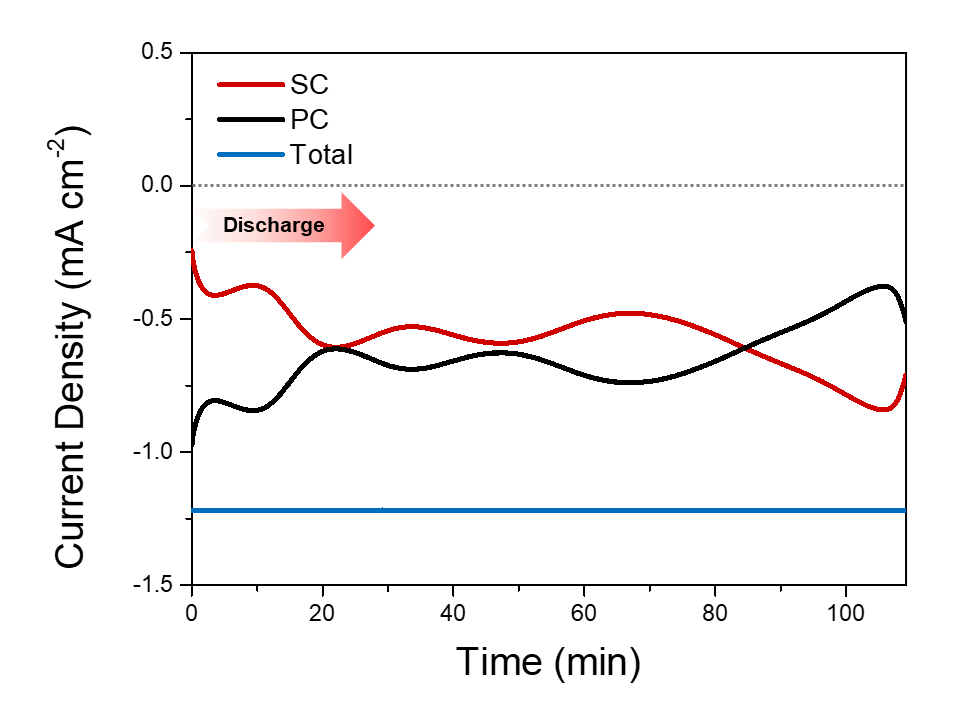

**Figure S3.** Measured current distribution profile of PCRD cell during 0.5C discharge


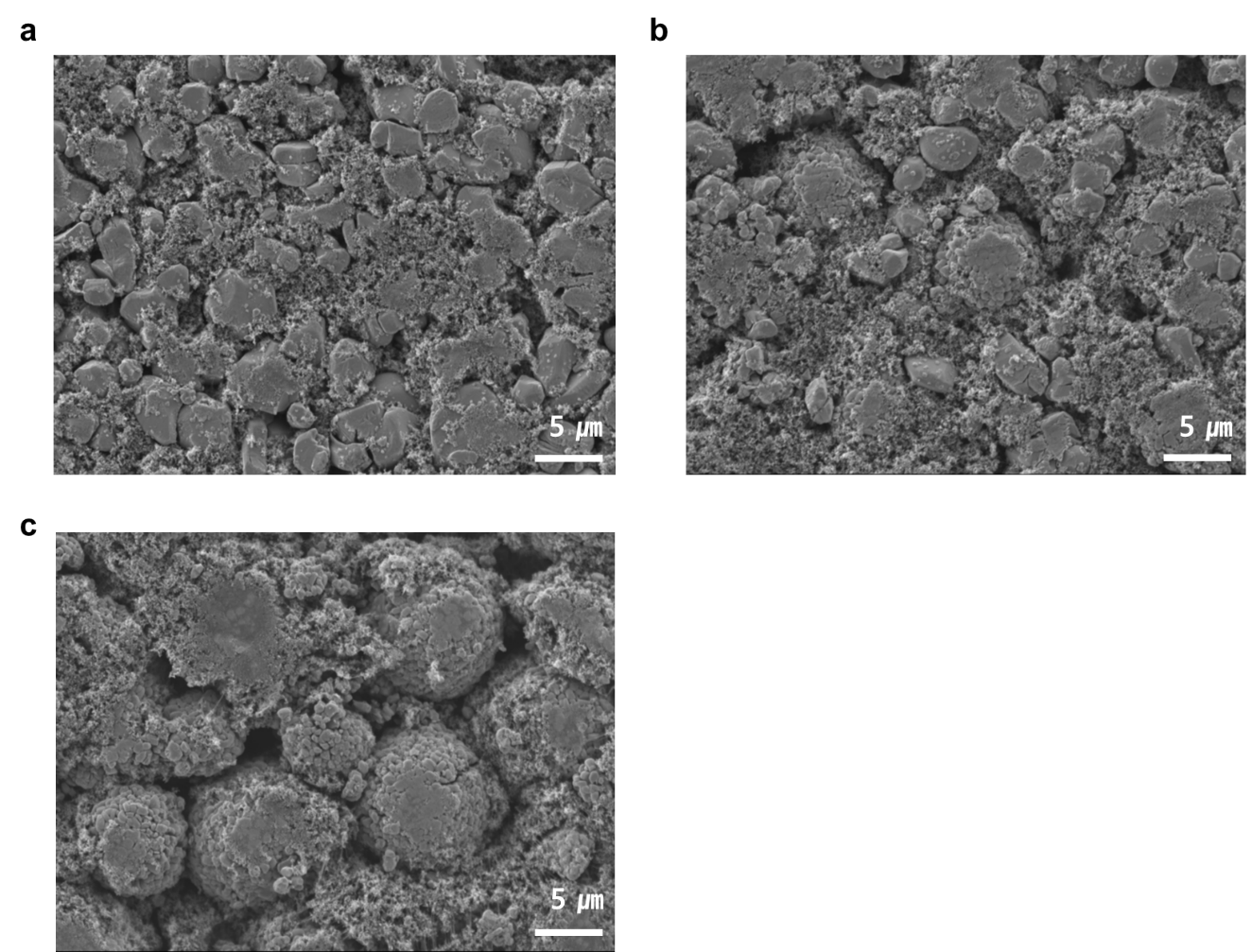


**Figure S4.** Top-view SEM images of as-prepared NCM811 electrodes depicting a) SC NCM811, b) bimodal NCM811, and c) PC NCM811.


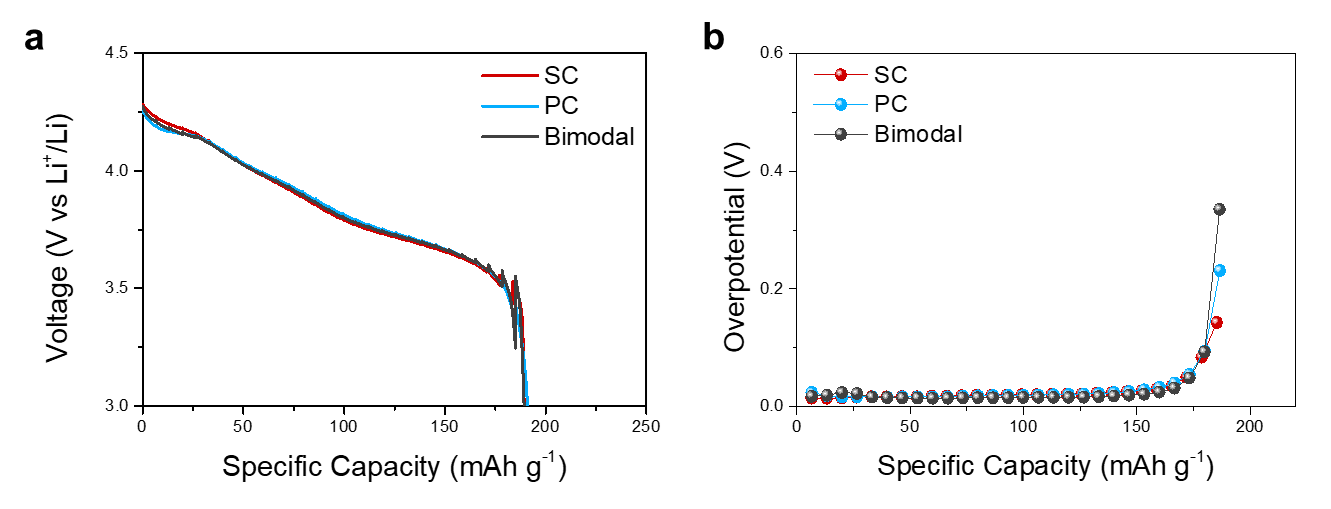


**Figure S5.** Electrochemical reaction overpotential comparison for SC, PC, and bimodal cathodes during discharge featuring e) GITT voltage profiles of Li | cathode half-cells at 0.5 C and f) overpotential profiles derived from the GITT data.


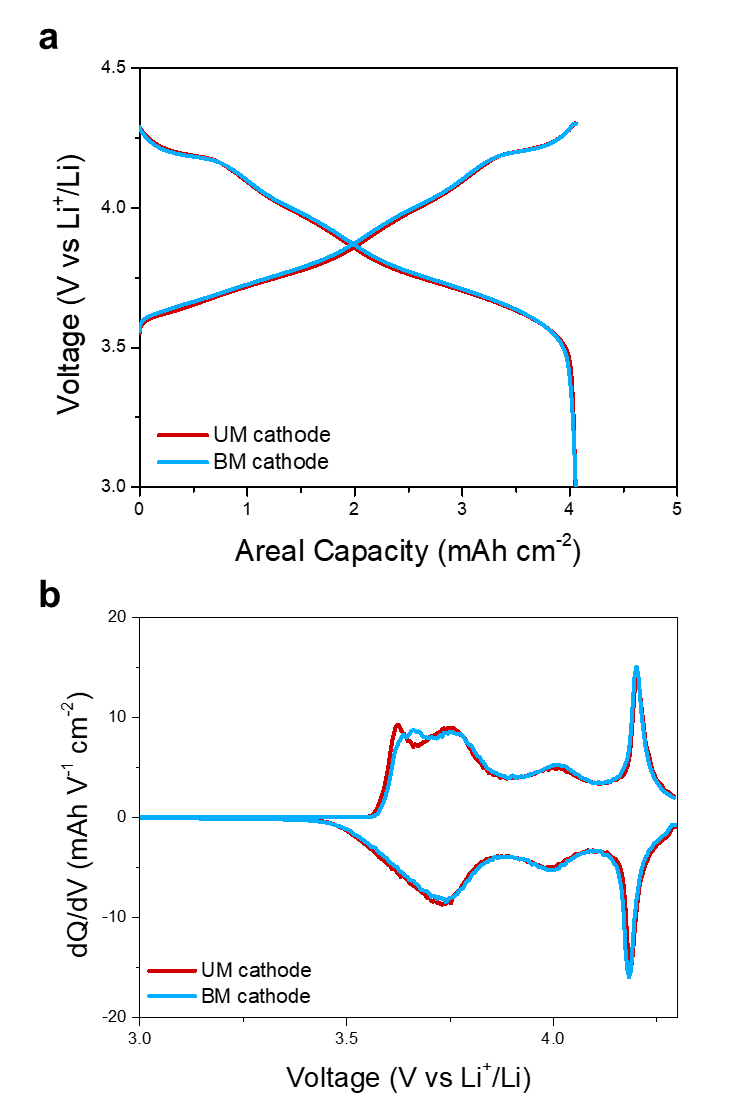


**Figure S6.** Electrochemical test of UM and BM cathode half-cells at 0.1 C presenting a) charge–discharge voltage profiles and b) differential capacity (dQ/dV) plots.


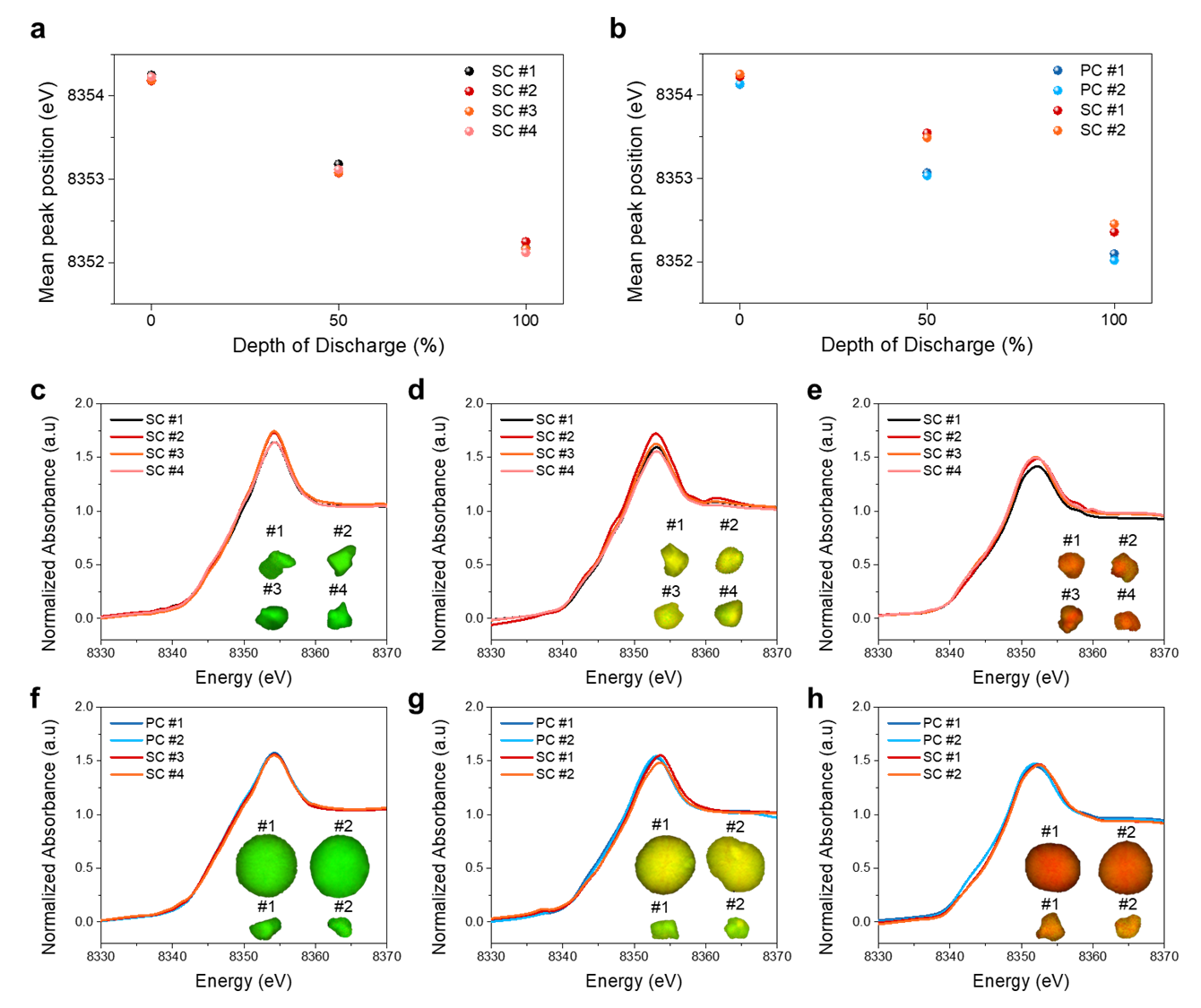


**Figure S7.** TXM–XANES analysis of UM and BM cathodes versus depth of discharge (DoD). Evolution of Ni K-edge mean peak positions for particles in a) UM cathode and b) BM cathode. Normalized Ni K-edge spectra for the UM cathode at c) 0 % DoD, d) 50 % DoD, e) 100 % DoD, and for the BM cathode at f) 0 % DoD, g) 50 % DoD, h) 100 % D nmoD.


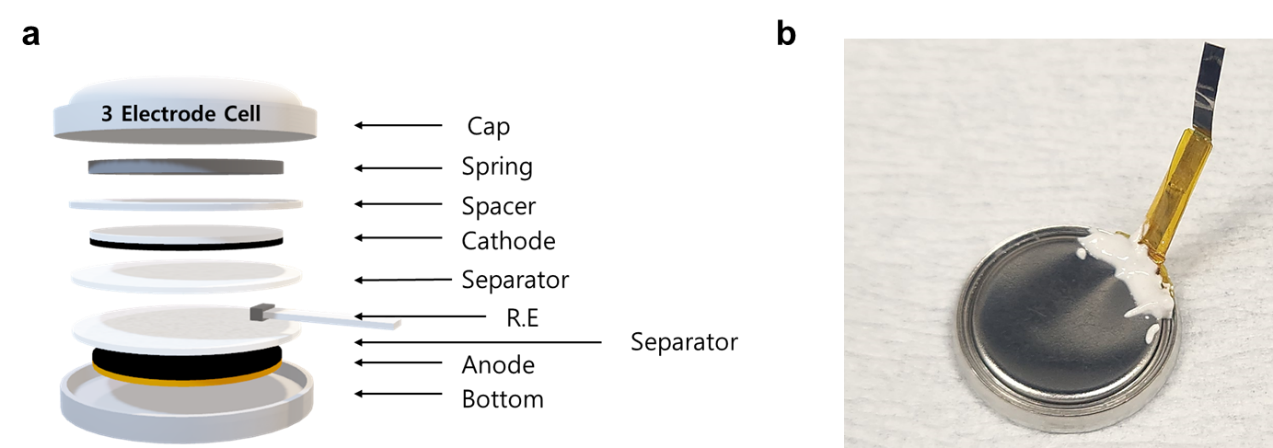


**Figure S8.** Configuration of the in-house assembled three-electrode coin cell system. a) schematic illustration of the cell structure with the reference electrode placed between two separators to prevent short-circuit and b) photograph of the assembled cell with external reference connection for accurate potential measurements during electrochemical testing.


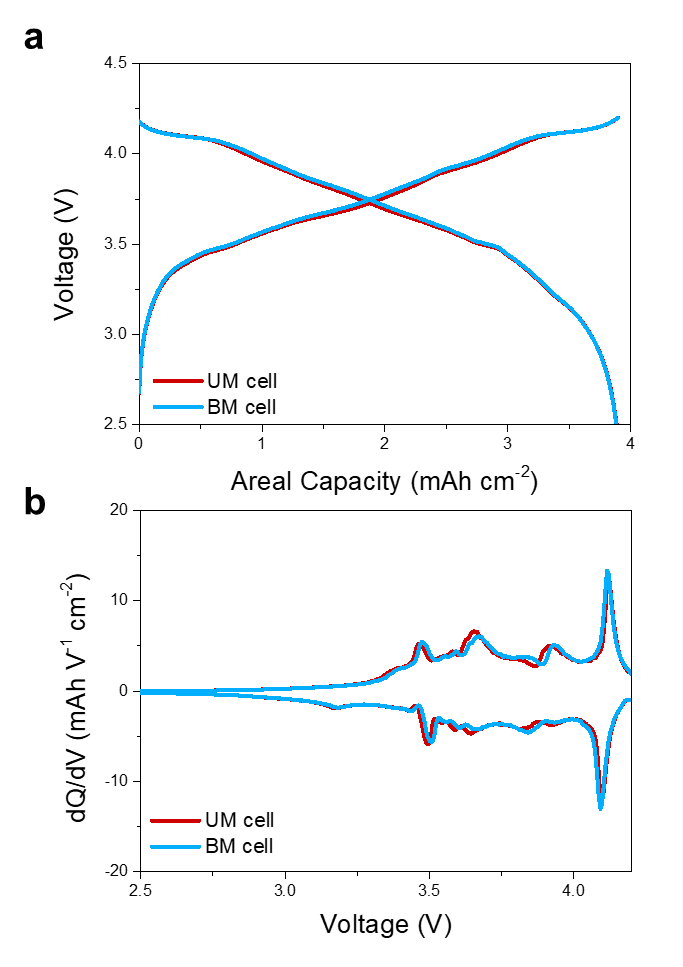


**Figure S9.** Electrochemical full-cell test of UM and BM cells at 0.05 C presenting a) charge-discharge voltage profiles and b) differential capacity (dQ/dV) plots.


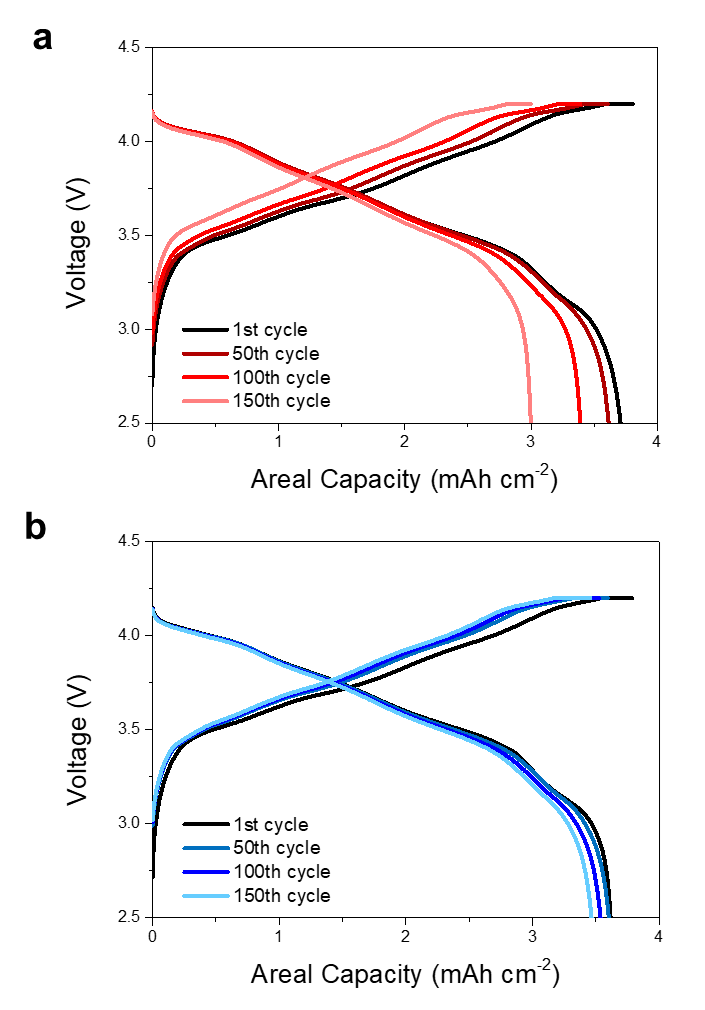


**Figure S10.** Charge-discharge voltage profiles of full cells at 0.33 C charge and 0.5 C discharge for a) UM cell and b) BM cell at the 1^st^, 50^th^, 100^th^, and 150^th^ cycles.


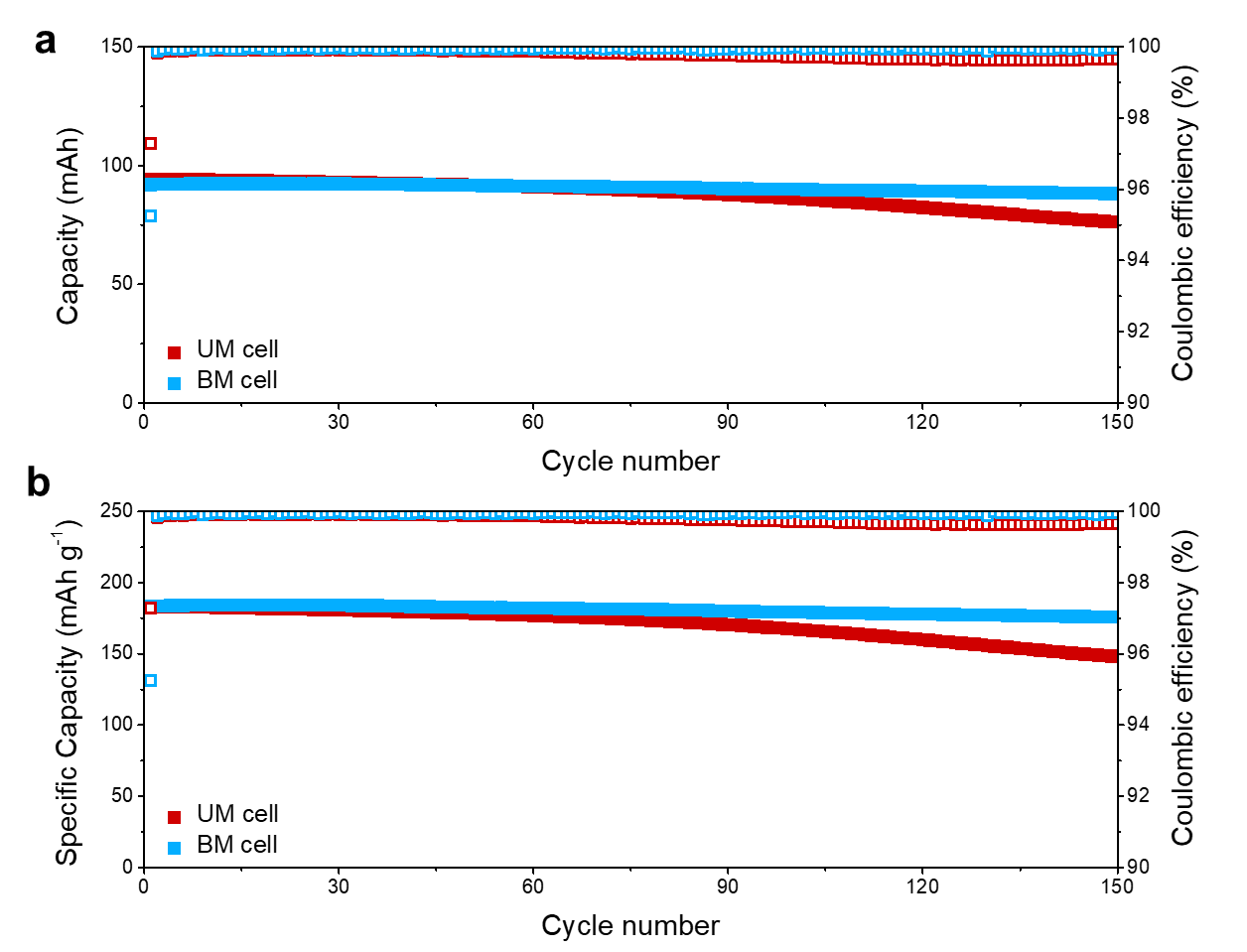


**Figure S11.** Cycling performance of the UM and BM pouch cells, plotted as a) actual capacity, b) specific capacity (mAh g⁻^1^)


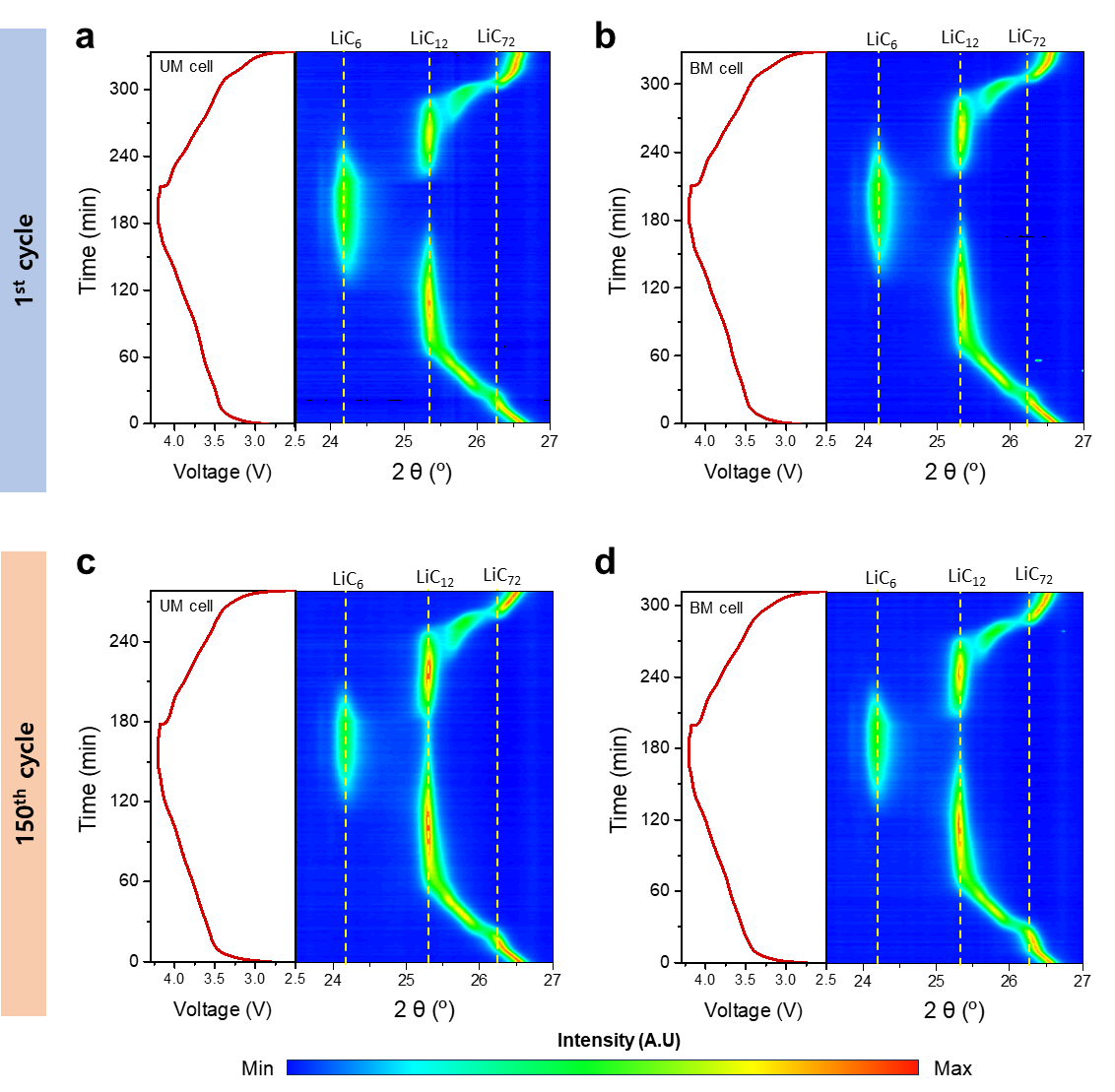


**Figure S12.** Operando XRD contour plots of graphite with corresponding voltage-capacity curves for a) UM cell and b) BM cell during the 1^st^ cycle, and c) UM cell and d) BM cell during the 150^th^ cycle.

**
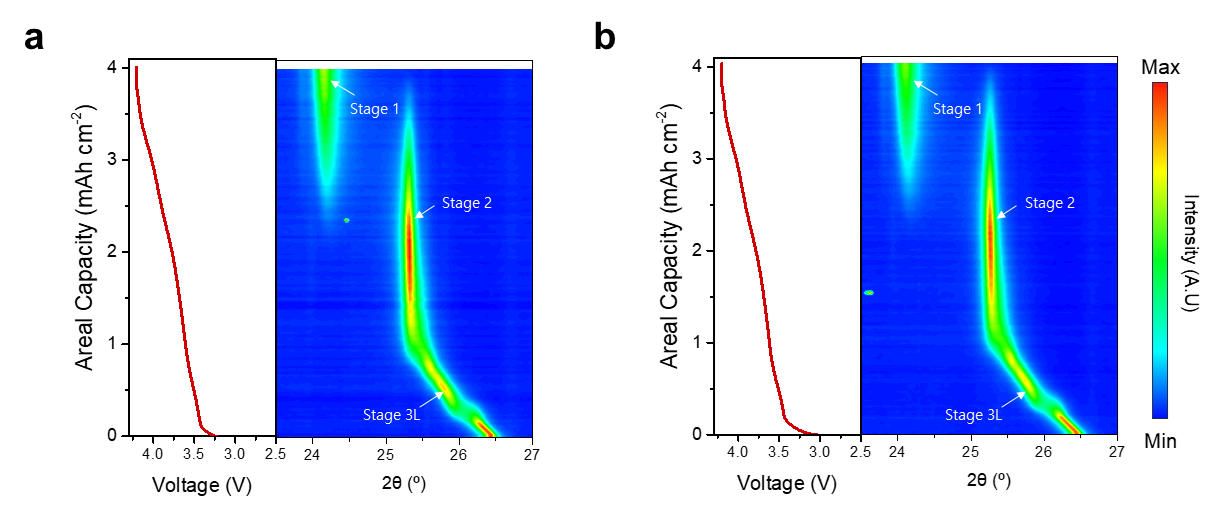
**

**Figure S13.** Operando XRD contour plots of graphite with corresponding voltage-capacity curves during 0.33 C charge for a) Graphite | UM cell and b) Graphite | BM cell at the 1^st^ cycle.


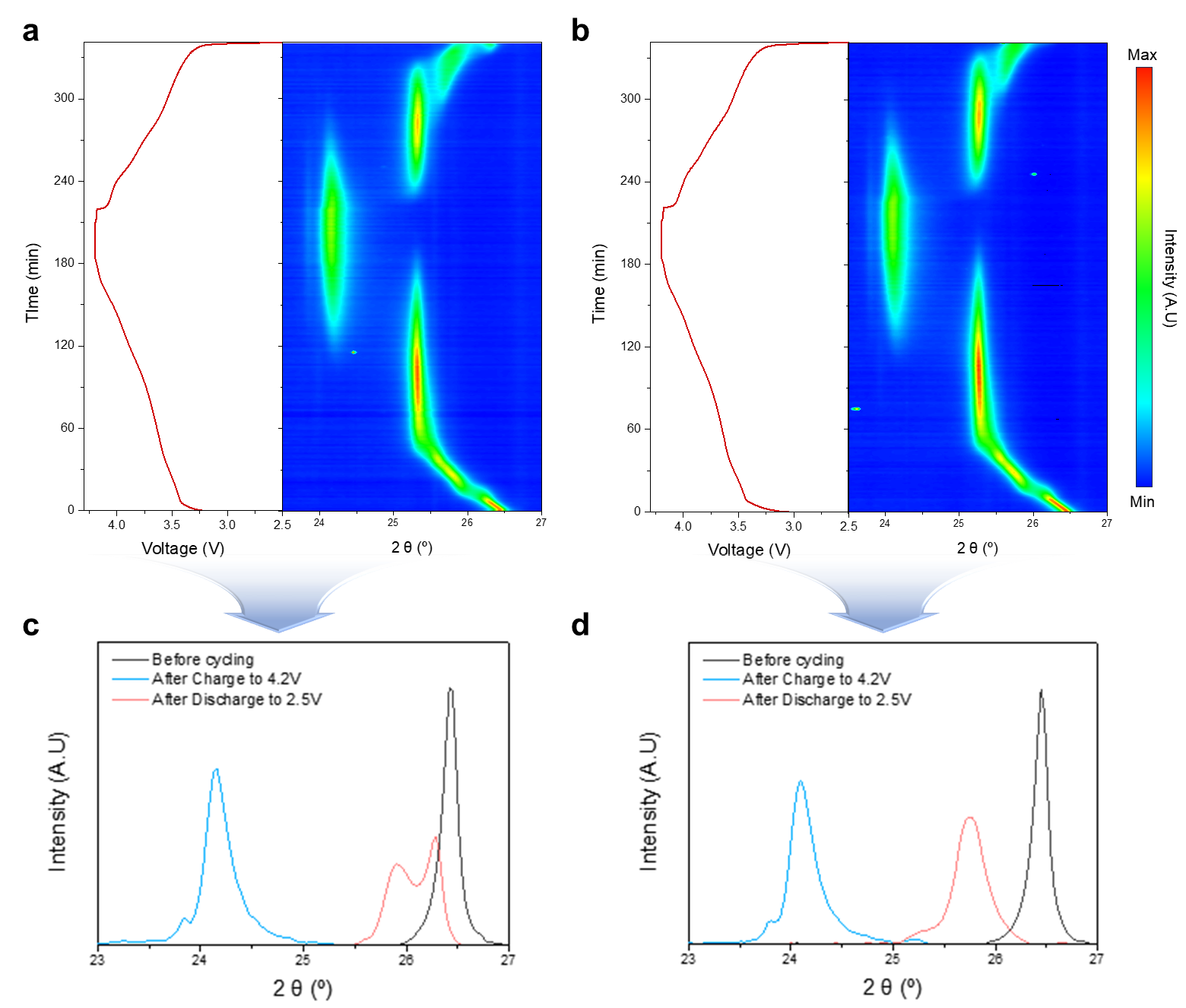


**Figure S14.** Operando XRD contour plots of graphite with corresponding voltage-capacity curves for a) Graphite | UM cell and b) Graphite | BM cell, and XRD patterns extracted at specific SoC states (before cycling, fully charged to 4.2 V, and fully discharged to 2.5 V) for c) Graphite | UM cell and d) Graphite | BM cell.

In the operando XRD profiles of the first cycle for full cells with graphite electrodes, the graphite peak of the BM cell is positioned at a lower two-theta angle compared to the UM cell when discharged to 2.5 V. This result aligns with previous electrochemical evaluations, indicating that the increased overpotential at the end of discharge in the BM cathode effectively suppresses de-lithiation in the anode.


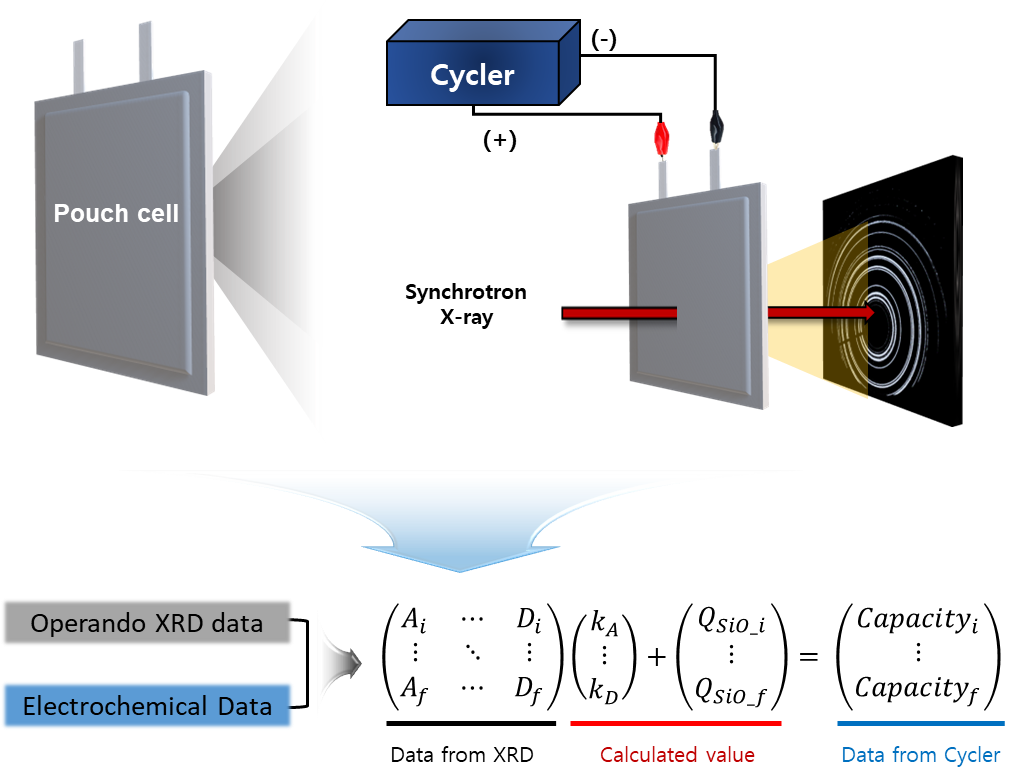


**Figure S15.** Schematic illustration of the operando XRD analysis experiment and the quantification process for lithium content in each active material of the SiO/Gr electrode.


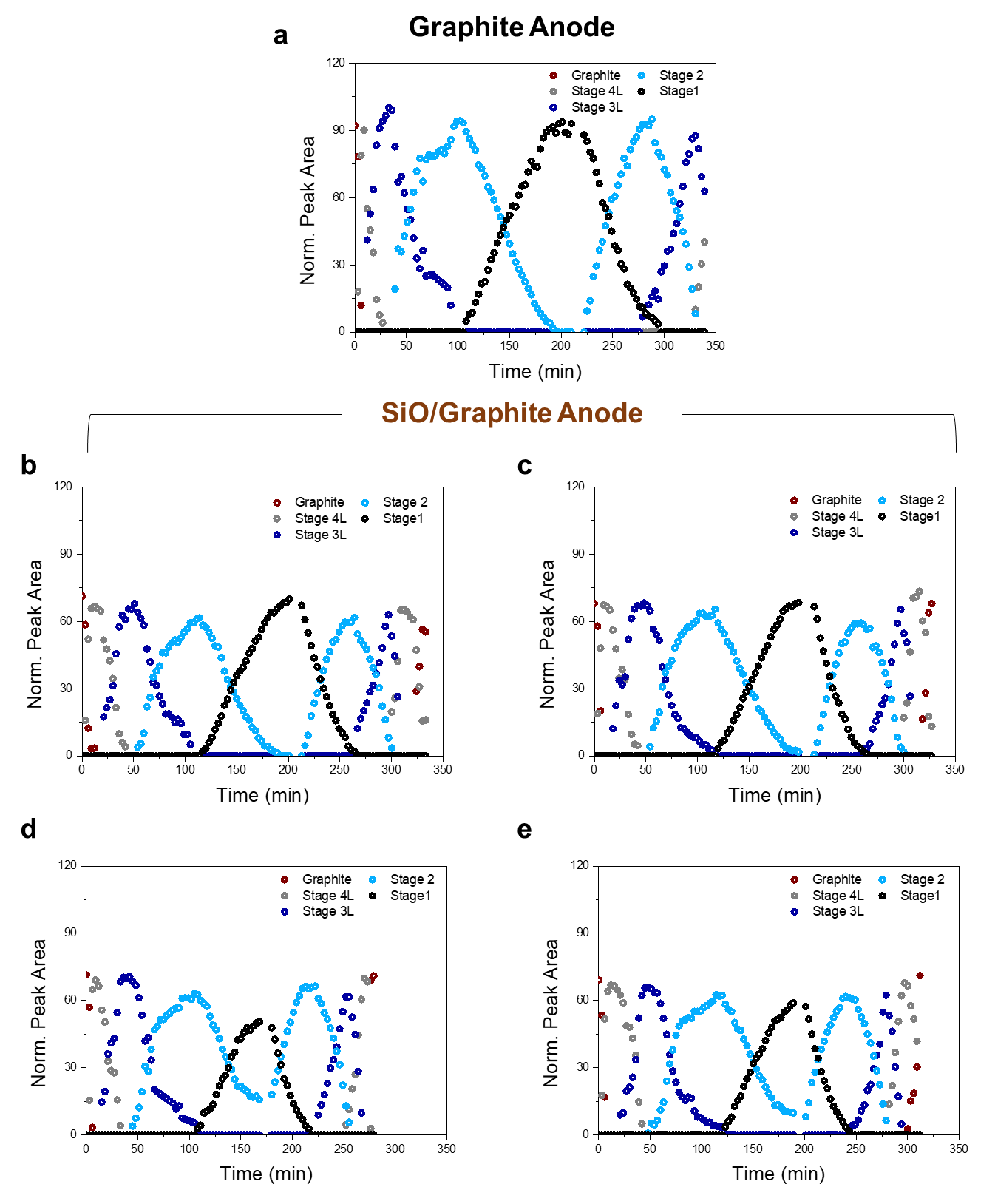


**Figure S16.** Deconvolution results for the diffraction peak of graphite with a) normalized peak area profiles of Li-graphite stages in the Graphite | NCM full cell, b) and c) UM cell at the 1^st^ and 150^th^ cycles, and d) and e) BM cell at the 1^st^ and 150^th^ cycles.


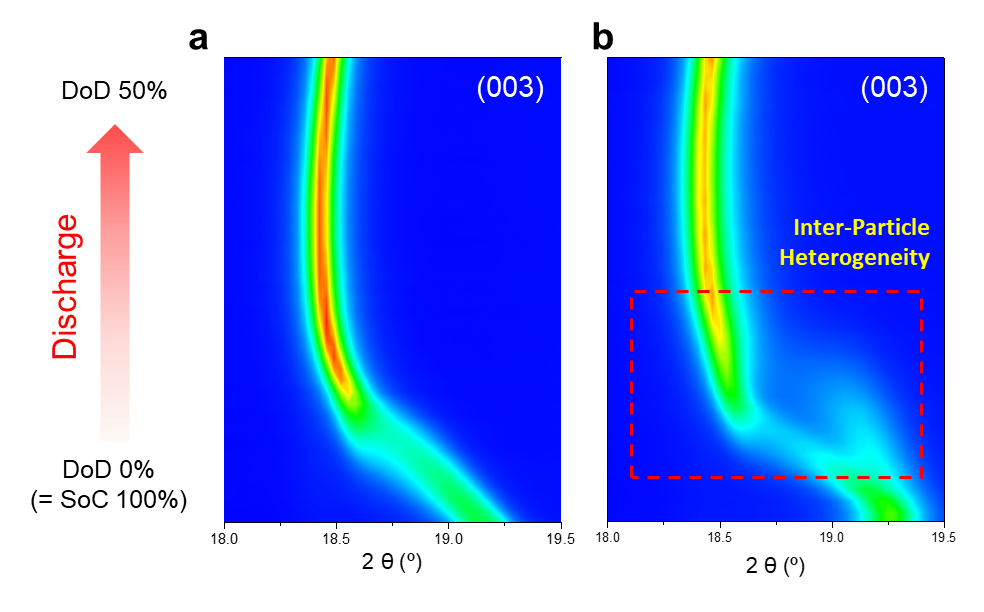


**Figure S17.** Phase evolution of cathodes during 0.5 C discharge with operando XRD contour plots of the (003) diffraction peaks from 0 to 50 % DoD for a) UM cathode and b) BM cathode.

**12**


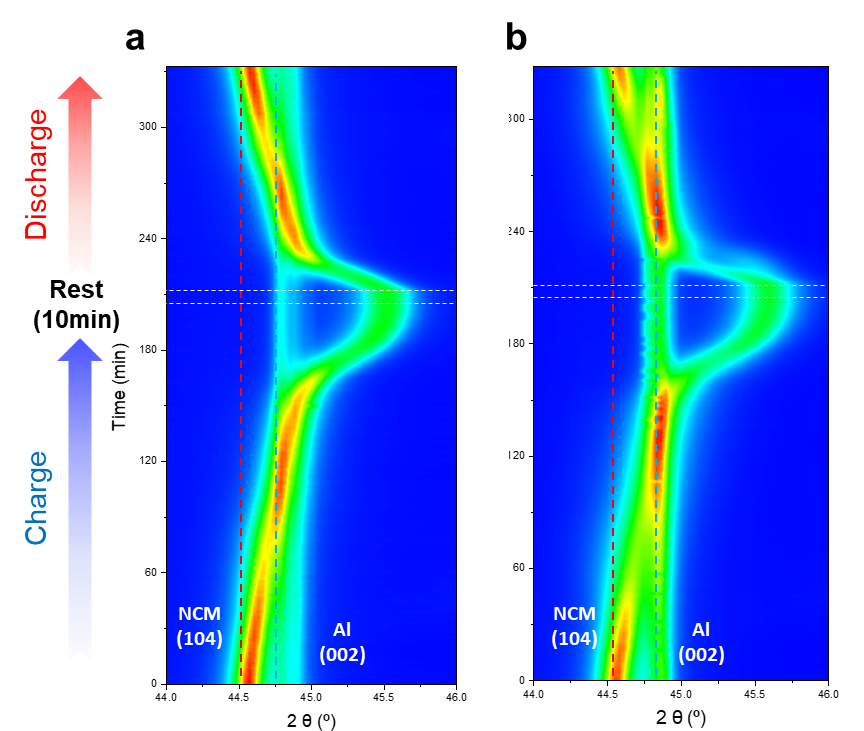


**Figure S18.** Operando XRD contour plots of the (104) diffraction peaks for a) UM cathode and b) BM cathode during charge–discharge.


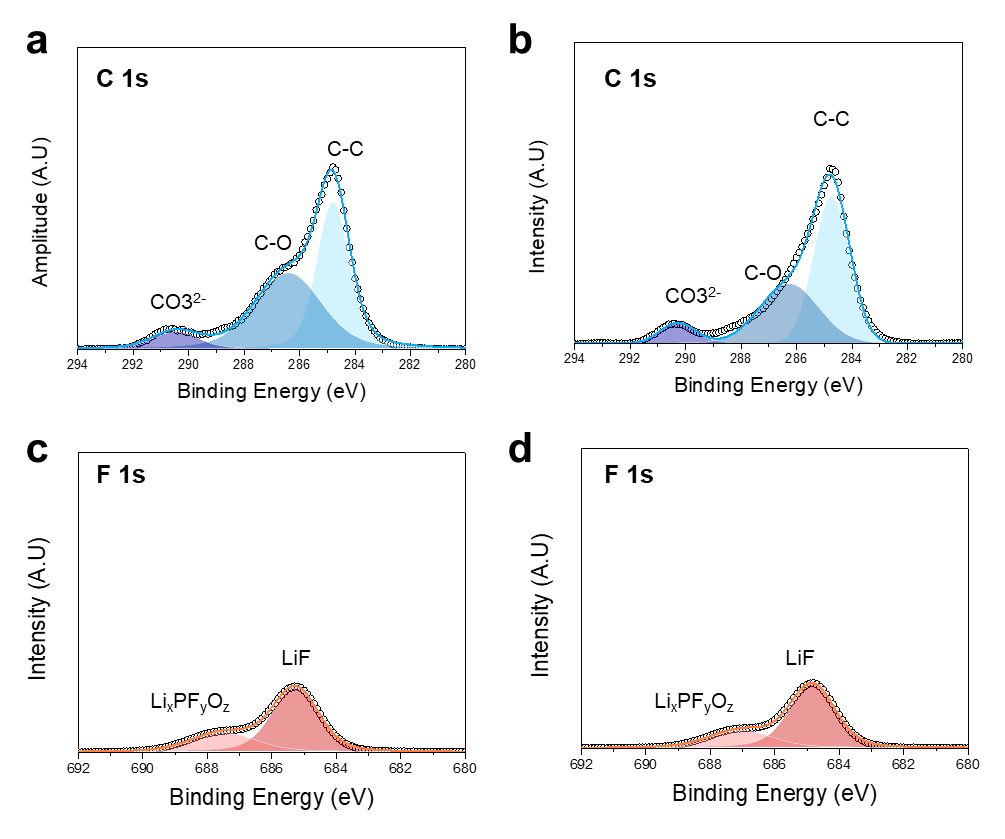


**Figure S19.** XPS spectra of SiO/Gr anodes after 1 cycle. C 1s spectra for a) UM cell and b) BM cell. F 1s spectra for c) UM cell and d) BM cell


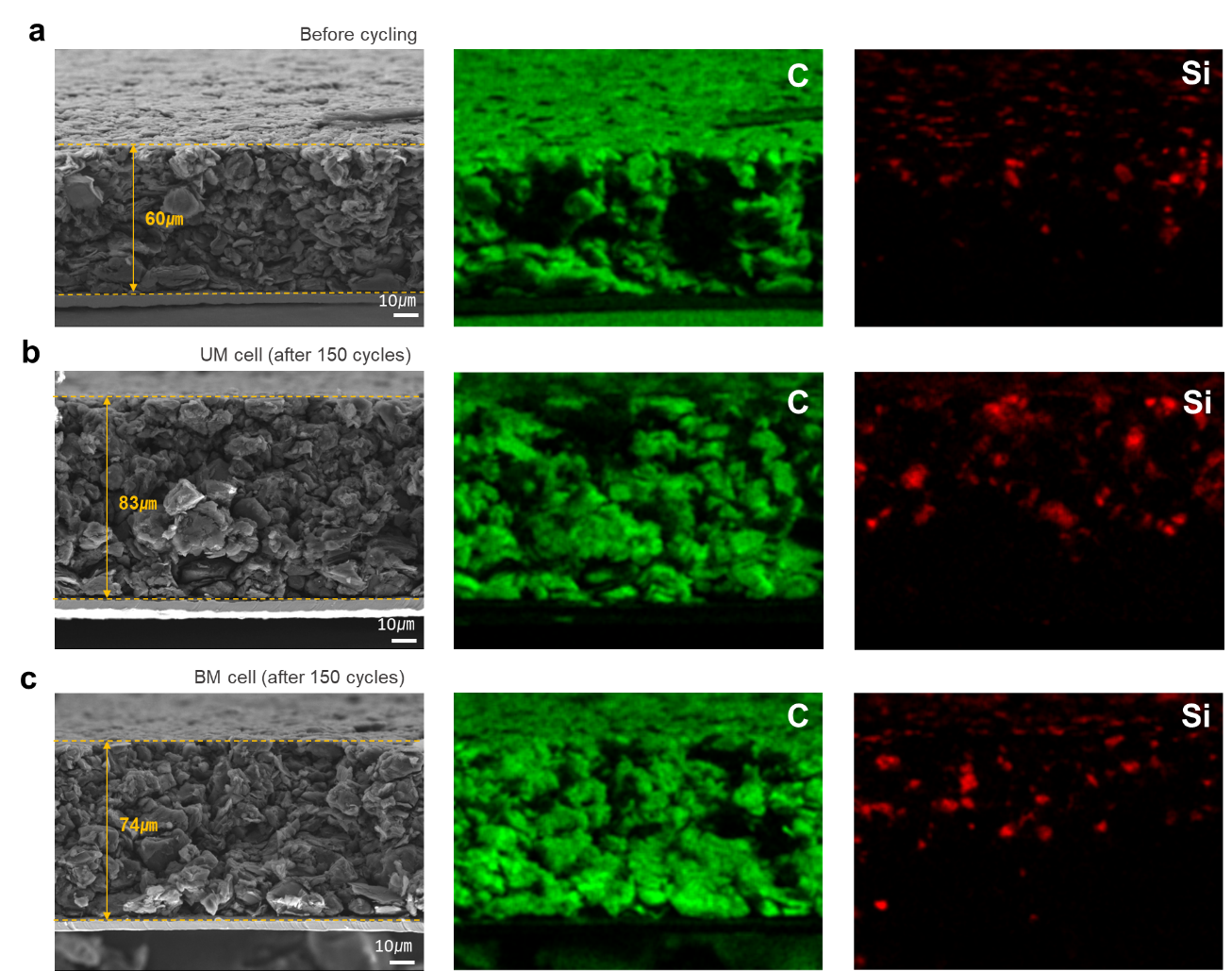


**Figure S20.** Cross-sectional SEM images and EDS elemental maps of SiO/Gr electrodes with a) electrode before cycling, and electrodes after 150 cycles from b) UM cell and c) BM cell.


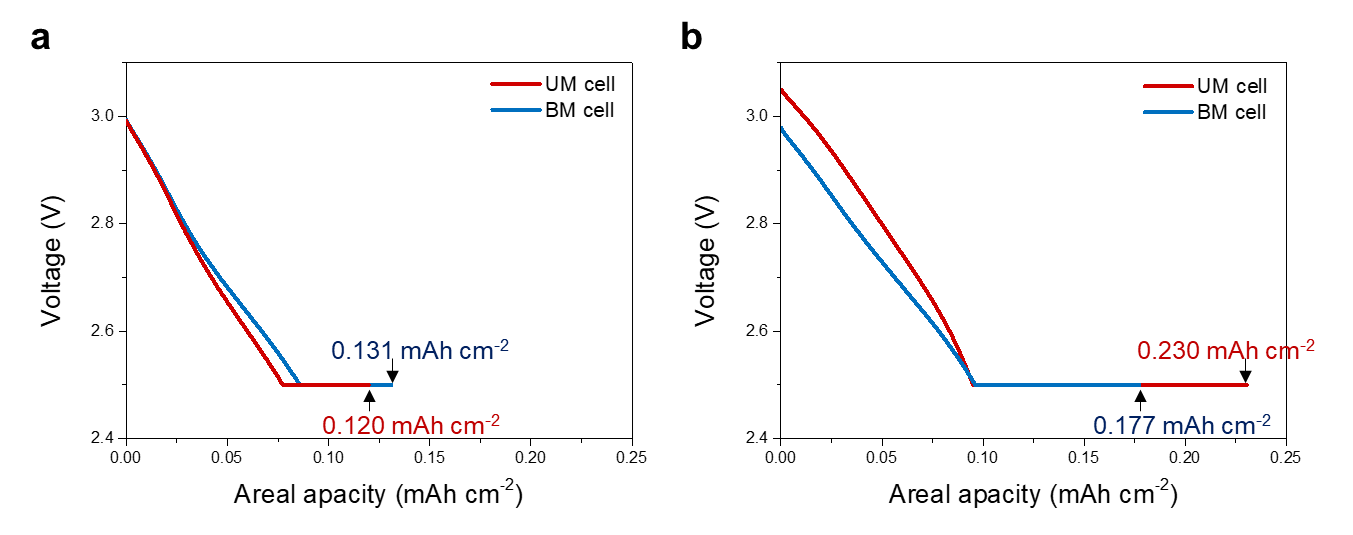


**Figure S21.** Voltage profiles of CC discharge (0.05 C) followed by CV discharge (2.5 V, 10 h) performed after a) 50 cycles and b) 150 cycles to evaluate residual active lithium in the SiO/Gr anode.


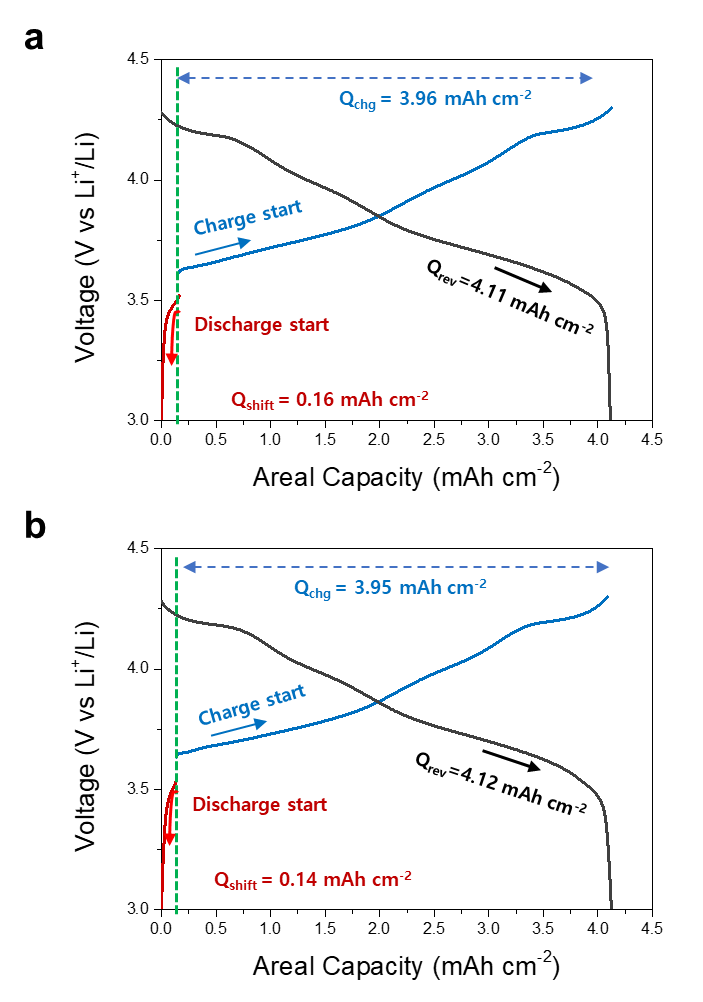


**Figure S22.** Voltage profiles of reassembled coin half-cells using a) UM cathode and b) BM cathode, with cells starting with discharge (red), starting with charge (blue), and subsequent discharge following charge (black).


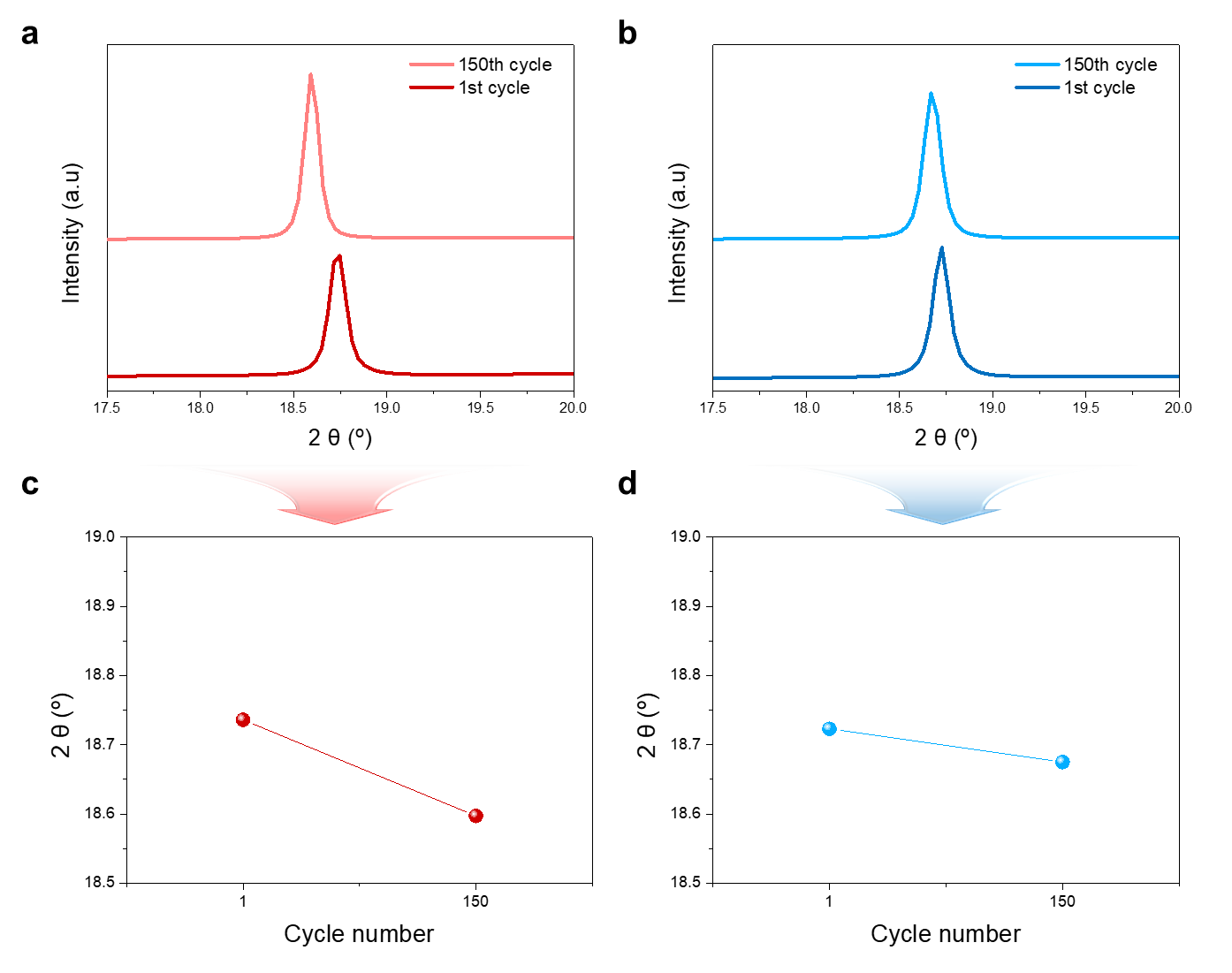


**Figure S23.** XRD patterns of the (003) diffraction peak at the 1^st^ and 150^th^ cycles for a) UM cathode and b) BM cathode, and fitted peak positions for c) UM cathode and d) BM cathode.


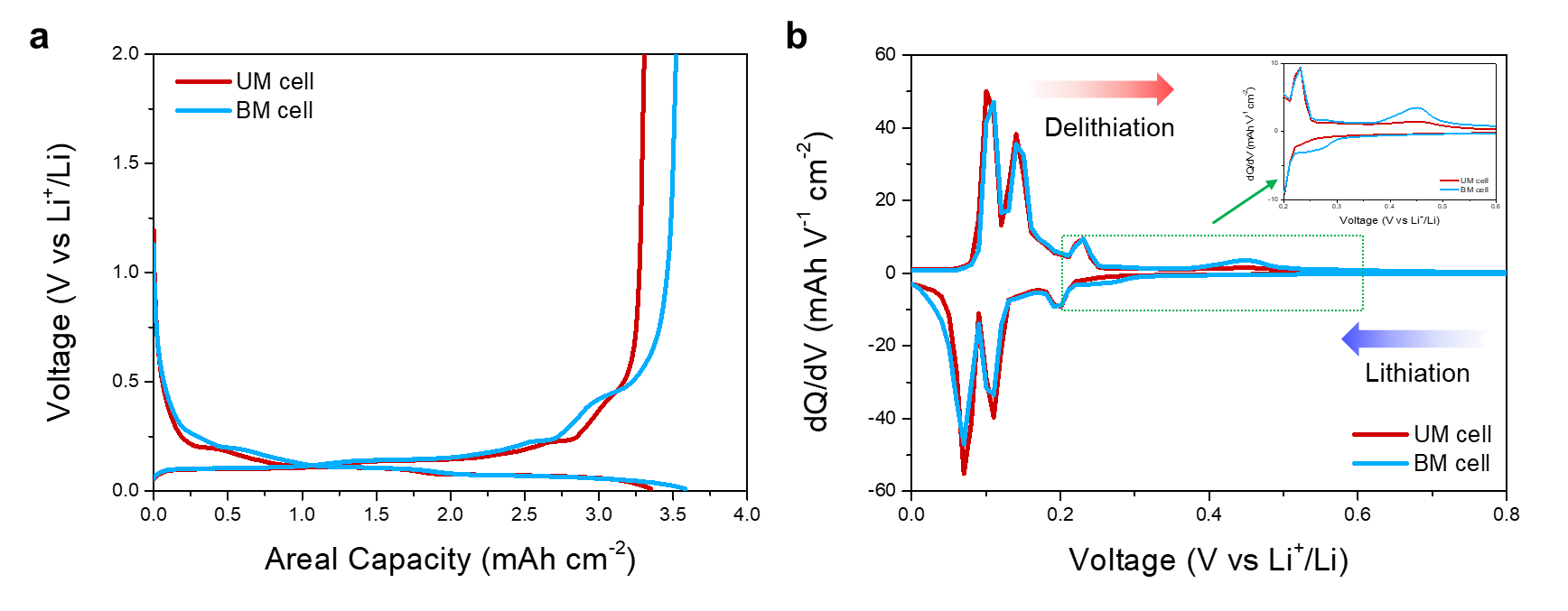


**Figure S24.** a) Charge–discharge voltage profiles and b) differential capacity (dQ/dV) plots of coin half-cells using SiO/Gr anodes collected from pouch cells after 150 cycles.


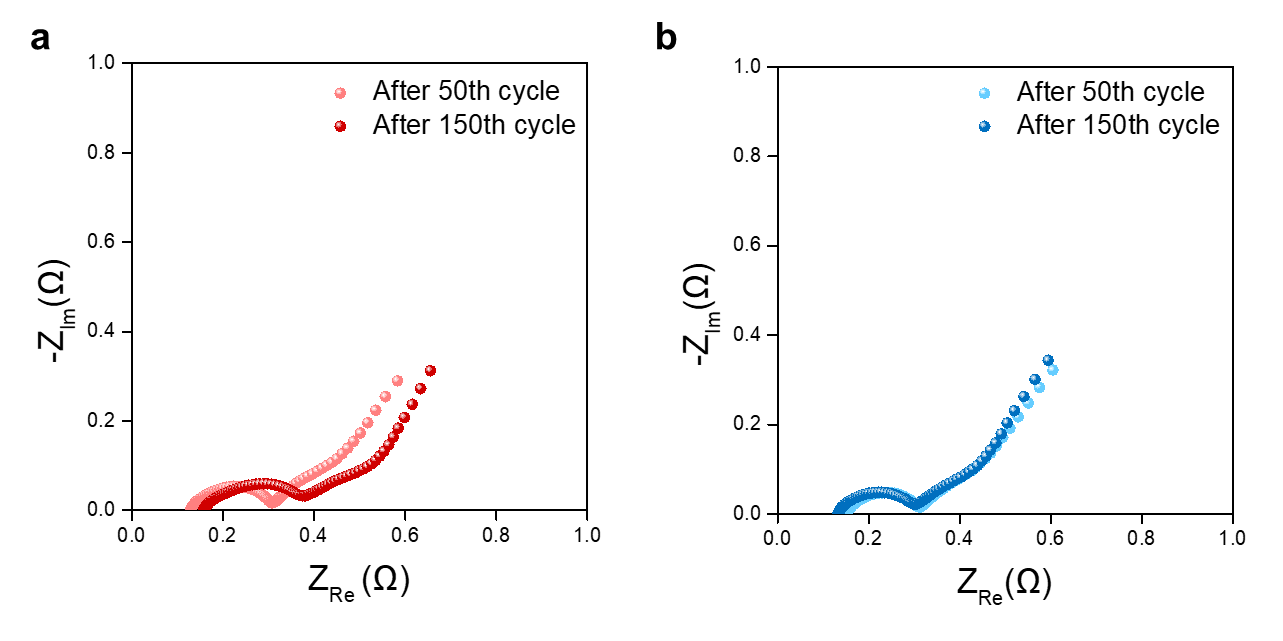


**Figure S25.** EIS Nyquist plots of full cells measured after the 50^th^ and 150^th^ cycle for a) UM cell and b) BM cell.


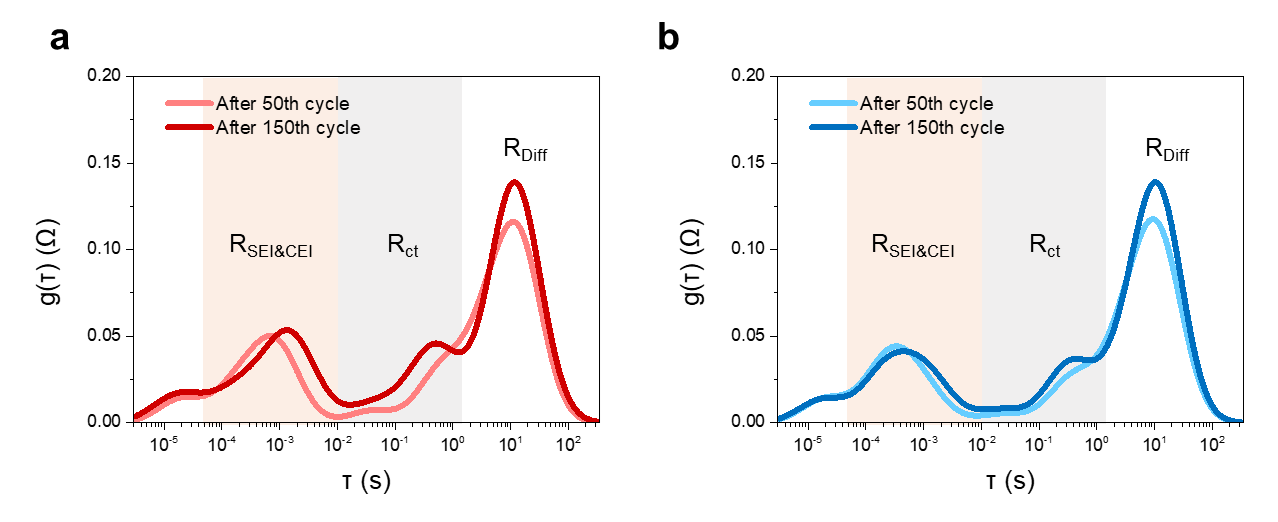


**Figure S26.** DRT plots calculated from the EIS data of full cells after the 50^th^ and 150^th^ cycle for a) UM cell and b) BM cell.


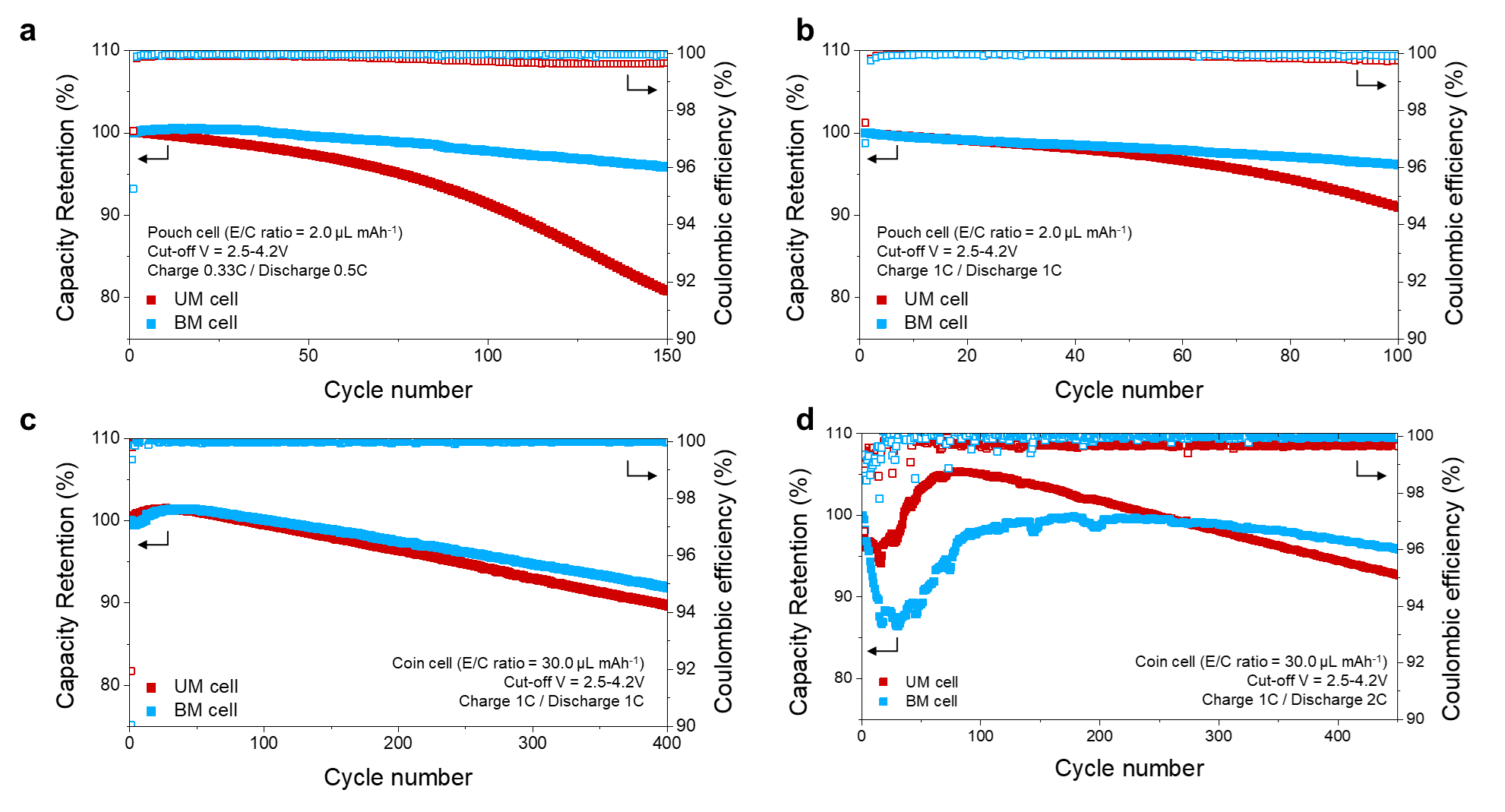


**Figure S27.** Cycling performance of the UM and BM cells in a,b) pouch cells and c,d) coin cells.

**Table S1. Calculation details of the gravimetric energy density of the pouch-type full cell based on electrodes**

|  | **Cu foil**  **(mg)** | **Anode mass loading**  **(mg cm^-2^)** | | **Anode**  **Area**  **(cm ^2^)** | **Al foil**  **(mg)** | **Cathode mass loading**  **(mg cm^-2^)** | **Cathode**  **Area**  **(cm ^2^)** | **Total**  **electrode**  **mass**  **(mg)** | **Q**  **(mAh)** | **Discharge V_ave_**  **(V)** | **Gravimetric energy density**  **(Wh kg^-1^)** |
| --- | --- | --- | --- | --- | --- | --- | --- | --- | --- | --- | --- |
| **UM** | 73 | | 10.5 | 26.66 | 165 | 20.3 | 25.20 | 1029 | 97.95 | 3.67 | 349.4 |
| **BM** |  |  |  |  |  | 19.7 |  | 1014 | 97.90 | 3.67 | 354.3 |

The gravimetric energy density (*E*, Wh kg⁻¹) was calculated using the following equation:

$$E=\frac{Q\times V_{ave}}{m_{total}}$$

where *Q* (mAh) is the discharge capacity, *V*ₐᵥₑ (V) is the average discharge voltage, and *m*ₜₒₜₐₗ (g) is the total mass of electrodes.

**Table S2.** Initial charge/discharge capacities and coulombic efficiencies for UM cell and BM cell

| **Cell type** | **Cell number** | **Charge Capacity**  **[mAh cm^-2^]** | **Average Charge Capacity**  **[mAh cm^-2^]** | **Discharge capacity**  **[mAh cm^-2^]** | **Average Discharge Capacity**  **[mAh cm^-2^]** | **Coulombic efficiency**  **[%]** | **Average**  **C.E**  **[%]** |
| --- | --- | --- | --- | --- | --- | --- | --- |
| UM cell | 1 | 3.84 | 3.85 | 3.74 | 3.75 | 97.22 | 97.18 |
|  | 2 | 3.87 |  | 3.76 |  | 97.04 |  |
|  | 3 | 3.85 |  | 3.74 |  | 97.28 |  |
| BM cell | 1 | 3.86 | 3.84 | 3.67 | 3.67 | 95.10 | 95.44 |
|  | 2 | 3.84 |  | 3.69 |  | 95.97 |  |
|  | 3 | 3.83 |  | 3.65 |  | 95.26 |  |
